# Multi-antigen coronavirus vaccine induces potent and durable cross-protection against multiple SARS-CoV-2 variants

**DOI:** 10.1038/s41541-026-01549-y

**Published:** 2026-08-18

**Authors:** Swayam Prakash, Nisha R. Dhanushkodi, Afshana Quadiri, Hawa Vahed, Aziz A. Chentoufi, Pierre-Gregoire Coulon, Izabela Coimbra Ibraim, Assia El Babsiri, Delia F. Tifrea, Cesar J. Figueroa, Daniel Gil, Jeffrey B. Ulmer, Lbachir BenMohamed

**Affiliations:** 1 Laboratory of Cellular and Molecular Immunology, Gavin Herbert Eye Institute, School of Medicine, University of California, Irvine, Irvine, CA USA; 2 Department of Vaccines and Immunotherapies, TechImmune, LLC, University Lab Partners, Irvine, CA USA; 3 BSL-3 Laboratories, High Containment Core Facility, School of Medicine, University of California, Irvine, Irvine, USA; 4 Department of Pathology and Laboratory Medicine, School of Medicine, Irvine, CA USA; 5 Department of Surgery, Divisions of Trauma, Burns & Critical Care, School of Medicine, Irvine, CA USA; 6 Institute for Immunology, School of Medicine, University of California, Irvine, Irvine, CA USA

**Keywords:** Diseases, Immunology, Microbiology

## Abstract

The first-generation Spike-based COVID-19 vaccines reduced the risk of hospitalization, serious illness, and death from SARS-CoV-2 infections. However, waning immunity failed to prevent immune escape, resulting in multiple variants of concern (VOCs) and prolonging the pandemic. We hypothesize that next-generation CoV vaccines incorporating highly conserved SARS-CoV-2 T cell antigens would confer potent, broad, long-lasting cross-protective immunity against multiple VOCs. In this study, we identified 10 non-Spike antigens common and highly conserved across 8.7 million SARS-CoV-2 strains, prior VOCs, SARS-CoV-1, MERS-CoV, seasonal human cold CoVs, and animal CoVs. Seven antigens were preferentially recognized by CD8^+^ and CD4^+^ T cells from unvaccinated asymptomatic COVID-19 patients, irrespective of VOC. Three conserved antigens (NSP2, NSP14, Nucleoprotein) administered to golden Syrian hamsters: (*i*) induced high frequencies of lung-resident polyfunctional CXCR5^+^CD4^+^ T_FH_ cells, GzmB^+^CD4^+^/CD8^+^ cytotoxic T cells, and CD69^+^IFN-γ^+^TNFα^+^CD4^+^/CD8^+^ effector T cells; (*ii*) reduced morbidity, viral load, lung pathology, and COVID-19-like symptoms from various VOCs, including the pathogenic B.1.617.2 Delta and the recent KP.3 Omicron variants; (*iii*) improved protection conferred by spike-alone mRNA; and (*iv*) conferred protection that lasted for more than 1 year. This multi-antigen CoV vaccine could provide potent, broad-spectrum, and durable cross-protective immunity against current and future variants of concern in the clinic.

## Introduction

The coronavirus disease 2019 (COVID-19) pandemic has created one of the most significant global health crises in nearly a century^1–6^. The first-generation Spike-based COVID-19 vaccines have contributed to reducing the COVID-19 pandemic and mitigating symptoms and deaths from SARS-CoV-2 infection^7,8^. However, since early 2020, breakthrough infections due to continued transmission have led to the emergence of VOCs and surges in hospitalizations, illnesses, and deaths, straining the world’s healthcare systems^9,10^. Today, thousands of patients are still being hospitalized and dying every month from COVID-19^11^. At the same time, ~10–30% of infected individuals with mild to moderate infections and up to 50% of people with severe infections carry the burden of long COVID, a debilitating chronic disease that currently affects over 10 million people in the U.S. and 410 million people globally, with no approved treatments^12–15^. Hence, a superior next-generation vaccine is desperately needed.

In response to immune escape and waning immunity to Spike-based COVID-19 vaccines, these first-generation vaccines have been periodically updated to incorporate Spike mutations from emerging variants that continue to emerge^16^. This vaccine strategy of chasing the variants and sub-variants cannot keep pace with the fast-emerging and rapidly mutating Omicron lineages^16^, as the sequences of the Spike protein, such as in the recently circulating Omicron subvariants KP.3, NB.1.8.1, and LP.8.1 Omicron subvariants, have already undergone over 100 accumulated mutations, diverging from the XBB1.5-adapted bivalent vaccine^17–19^. As a consequence, the bivalent vaccine introduced in 2022 was only effective 4–29% of cases against the Omicron subvariants, circulating that winter^17–19^, and its effectiveness decreased even further against the more recent divergent and highly transmissible EG.5, HV.1, JN.1, JN.3, KP.3, NB.1.8.1, and LP.8.1 Omicron subvariants, circulating in the winters of 2024–2025^17–19^. These observations underscore the need for a superior next-generation CoV vaccine strategy that induces broad, durable, and cross-protective immunity^20–22^, thereby halting the ongoing COVID-19 pandemic and providing protection against future SARS-CoV-2 variants^23^.

Recently, our group and others have: (*i*) identified specific sets of highly conserved SARS-CoV-2 non-Spike antigens targeted by frequent cross-reactive functional CD4^+^ and CD8^+^ T cells from asymptomatic COVID-19 patients (i.e., unvaccinated individuals who never develop any COVID-19 symptoms despite being infected)^3,5,24–30^; (*ii*) discovered that increased frequencies of lung-resident CD4^+^ and CD8^+^ T cells specific to common CoV antigens protected against multiple SARS-CoV-2 VOCs in mouse models^1,3,31,32^; and (*iii*) demonstrated that enriched cross-reactive lung-resident memory CD4^+^ and CD8^+^ T_RM_ cells that selectively target early-transcribed SARS-CoV-2 antigens from the replication and transcription complex (RTC) region are associated with a rapid clearance of infection in so-called “SARS-CoV-2 aborters” (i.e., unvaccinated SARS-CoV-2 exposed seronegative individuals who rapidly abort the virus replication)^33–37^. We hypothesize that a next-generation CoV vaccine that incorporates highly conserved and early expressed RTC antigens selectively targeted by CD4^+^ and CD8^+^ T cells from asymptomatic COVID-19 patients and “SARS-CoV-2 aborters” would confer stronger, broader, and longer-lasting protective immunity against rapidly transmissible and highly pathogenic VOCs.

In the present study, using in silico bioinformatic techniques, we identified non-Spike RTC antigens that are highly conserved in 8.7 million genome sequences of SARS-CoV-2 strains that have circulated worldwide, including previously designated SARS-CoV-2 VOCs, SARS-CoV-1, MERS-CoV, seasonal common cold coronaviruses, and animal CoVs (i.e., bats, civet vats, pangolins, and camels). Seven non-Spike highly conserved antigens were selectively recognized by cross-reactive CD4^+^ and CD8^+^ T cells from unvaccinated asymptomatic COVID-19 patients. Three of these seven T cell antigens, when delivered as mRNA/LNP, safely induced superior B and T_RM_ cell cross-protective immunity compared to spike-alone mRNA vaccines, which lasted more than 12 months post-vaccination against several pathogenic and heavily mutated SARS-CoV-2 variants and sub-variants in the hamster model. These findings provide critical insights into the development of B and T cell antigen-based CoV vaccines that can confer broad and long-lasting, cross-protective immunity against highly mutated and pathogenic VOCs.

## Results

### Five highly conserved regions that encode 10 common structural, non-structural, and accessory protein antigens were identified in the SARS-CoV-2 genome

**Fig. 1:**
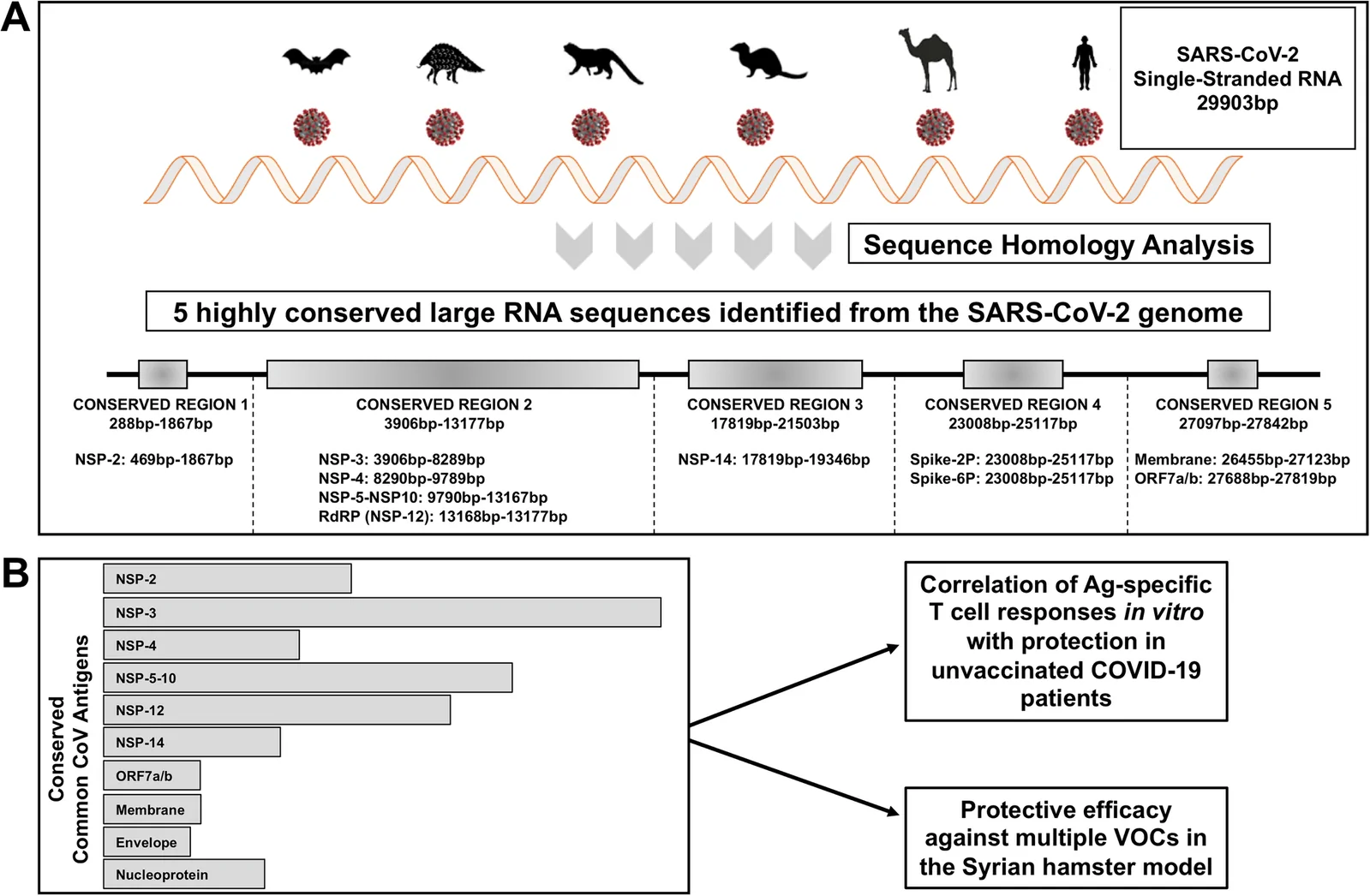
Highly conserved non-Spike, structural, non-structural, and accessory protein antigens identified in the SARS-CoV-2 genome. **A** Sequence homology analysis of 8.7 million genome sequences of SARS-CoV-2 strains that circulated worldwide over the last 5 years, including 20 VOCs; previously reported SARS-CoV, MERS-CoV, and common cold Coronaviruses; and 25 animal-specific SARS-like Coronaviruses (SL-CoVs) genome sequences isolated from bats (Rhinolophus affinis, Rhinolophus malayanus), pangolins (Manis javanica), civet cats (Paguma larvata), and camels (Camelus dromedaries). Shown in gradient light gray are five highly conserved regions identified in the SARS-CoV-2 genome sequences, along with the detailed segmentation and annotation of the conserved regions. **B** Highly conserved non-Spike antigens that comprise structural (Membrane), non-structural (NSP-2, NSP-3, NSP-4, NSP-5-10, NSP-12, and NSP-14), and accessory protein (ORF7a/b) as potential T cell antigens used to construct the individual and combined mRNA/LNP vaccines. We have also screened two additional structural proteins (Envelope and Nucleoprotein), for which we didn’t find significant sequence homology. The boxes illustrate the strategies used to evaluate the potential of these T-cell antigens to elicit protective immunity.

The SARS-CoV-2 single-stranded genome comprises 29,903 base pairs (bp), which encodes 29 proteins, including four structural, 16 non-structural, and nine accessory regulatory proteins^38^. We have used several in silico bioinformatic approaches to perform sequence alignments of 8.7 million genome sequences of SARS-CoV-2 variants that circulated worldwide throughout the pandemic, including all the previously designated SARS-CoV-2 variants of concern (VOC), variants of interest (VOI), and variants being monitored (VBM). This includes sequences of Coronavirus strains reported before the COVID-19 pandemic, like SARS-CoV-1; MERS-CoV; common cold coronaviruses (i.e., α-CCC-229E, α-CCC-NL63, β-CCC-HKU1, and β-CCC-OC43); and 25 enzoonotic SARS-like CoVs (SL-CoVs) genome sequences isolated from bats, pangolins, civet cats, and camels (Fig. 1). To overcome the overrepresentation of 8.7 million SARS-CoV-2 reads for sequencing homology analysis, which could lead to skewing the establishment of any kind of consensus of conservation, we have performed a small-scale sequence alignment comprising individual sequences each specific to unique SARS-CoV-2, SARS-CoV-1, MERS-CoV, CCC, and zoonotic SARS-like CoV strains. Based on the extensive sequence alignment analysis, we have identified five highly conserved regions in the SARS-CoV-2 single-stranded RNA genome (1–1580 bp, 3547–12,830 bp, 1772–21,156 bp, 22,585–24,682 bp, and 26,660–27,421 bp, Fig. 1). Further sequence homology analysis confirmed that the five SARS-CoV-2 genome regions encode for 10 highly conserved non-Spike T cell antigens (NSP-2 (size: 1914 bp, nucleotide range: 540–2454 bp), NSP-3 (size: 4485 bp, nucleotide range: 3804–8289 bp), NSP-4 (size: 1500 bp, nucleotide range: 8290–9790 bp), NSP-5-10 (size: 3378 bp, nucleotide range: 9791–13,169 bp), NSP-12 (size: 2796 bp, nucleotide range: 13,170–15,966 bp), NSP-14 (size: 1581 bp, nucleotide range: 17,766–19,347 bp), ORF7a/b (size: 492 bp, nucleotide range: 27,327–27,819 bp), Membrane (size: 666 bp, nucleotide range: 26,455–27,121 bp), Envelope (size: 225 bp, nucleotide range: 26,177–26,402 bp), and Nucleoprotein (size: 1248 bp, nucleotide range: 28,206–29,454 bp)) (Fig. 1). The gene sequences of these 10 highly conserved antigens were then used to design and construct N1-methyl-pseudouridine (m1ψ)-modified mRNAs encapsulated in lipid nanoparticles (mRNA/LNP) for assessment of safety, immunogenicity, and protective efficacy against several SARS-CoV-2 VoCs in the golden Syrian hamster model (Fig. 1).

Mutations screened against 12 major SARS-CoV-2 variants and sequence homology analysis confirmed the sequences representing the 10 non-Spike antigens are highly conserved in the currently highly mutated BA.2.86, JN.1, KP.3, NB.1.8.1, and LP.8.1 Omicron sub-variants. As expected, with 346 cumulative mutations since the ancestral Wuhan strain, the Spike sequence is highly mutated in the latest Omicron subvariants relative to the non-Spike antigens. The Spike protein sequences exhibit new mutations in the current highly transmissible and most immune-evasive Omicron subvariants. While the Spike protein of Omicron BA.2.86 has a total of 61 non-synonymous mutations, Omicron JN.1 and Omicron KP.3 have 62 and 65 Spike-specific non-synonymous mutations, respectively. In contrast, compared to Spike, the NSP-2 has two common mutations (A211D and P314L), and Nucleoprotein has eight common mutations shared among Omicron BA.2.86, JN.1, and KP.3 variants (P13L, E31-, R32-, S33-, R203K, G204R, Q229K, S413). No non-synonymous mutation in NSP-14 has been reported for any of these Omicron subvariants. Notably, the NSP-14 sequence is fully conserved (100%) across all variants and subvariants, including recent variants JN.1, KP.3, NB.1.8.1, and LP.8.1, supporting the vital role of these antigens in the life cycle of SARS-CoV-2. Of the 10 non-Spike antigens, Nucleoprotein was the least conserved in all variants and sub-variants, but remains an important vaccine target, as it is the most abundant viral protein and one of the most predominantly targeted antigens by T cells in individuals with less severe COVID-19 disease^39,40^.

### Human memory CD4^+^ and CD8^+^ T cells preferentially target seven of the 10 highly conserved SARS-CoV-2 antigens and correlate with improved disease outcome in unvaccinated asymptomatic COVID-19 patients

We next determined whether the 10 highly conserved non-Spike antigens are targeted by CD4^+^ and CD8^+^ T cells from “naturally protected” unvaccinated COVID-19 patients.

**Fig. 2:**
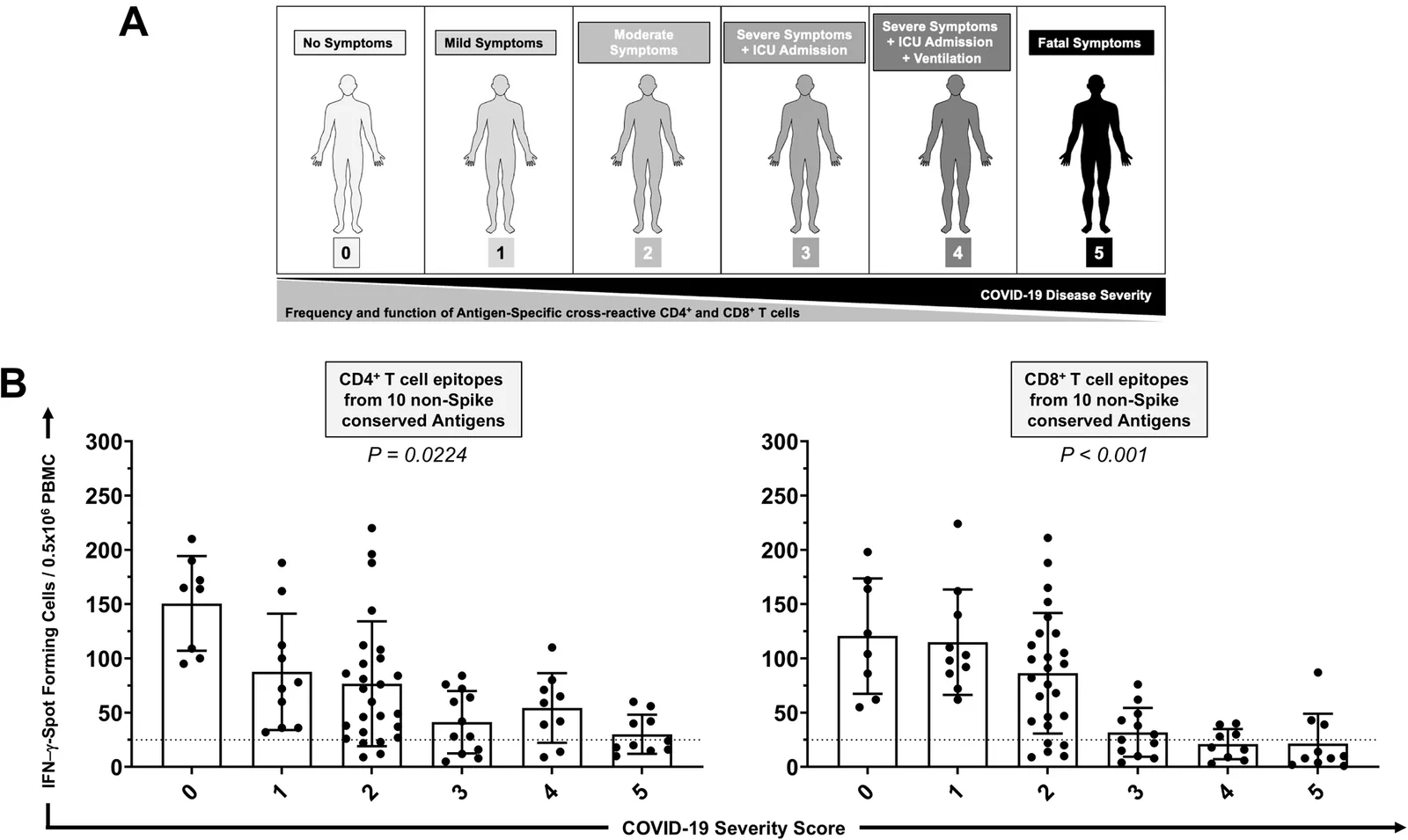
IFN-γ-producing CD4^+^ and CD8^+^ T cell responses to highly conserved antigens in unvaccinated COVID-19 patients with various degrees of disease severity. **A** The degrees of severity of COVID-19 disease in unvaccinated COVID-19 patients (*n* = 71), as divided into six groups, scored 0 to 5 and described in “Methods” (black = severity 5, to white = severity 0). **B** PBMCs from HLA-DR- and HLA-A*0201-positive COVID-19 patients were isolated and stimulated for a total of 72 h with a pool of peptides corresponding to CD4^+^ and CD8^+^ T cell epitopes from the 10 selected conserved antigens (i.e., NSP-2, NSP-3, NSP-4, NSP-5-10, NSP-12, NSP-14, ORF7a/b, Membrane, Envelope, and Nucleoprotein). The number of IFN-γ-producing T cells was quantified in each of the 71 patients using an ELISpot assay. Shown are the average/mean numbers (±SD) of IFN-γ-spot forming cells (SFCs) for CD4^+^ (*left panel*) or CD8^+^ (*right panel*) T cell responses divided into six groups based on disease severity. PHA was used as a positive control of T-cell activation. Unstimulated negative control SFCs were subtracted from the SFC counts of peptide-stimulated cells. For all graphs, the coefficient of determination (*R*^2^) is calculated from the Pearson correlation coefficients. The associated *P* value and the slope (S) of the best-fitted line (dotted line) are calculated using linear regression analysis and are indicated. The gray-hatched boxes in the correlation graphs extend from the 25th to the 75th percentiles (hinges of the plots), with the median represented as a horizontal line in each box, and the extremities of the vertical bars showing the minimum and maximum values. Results are representative of two independent experiments and were considered statistically significant at *P* ≤ 0.05 using either the Mann–Whitney test (two groups) or the Kruskal–Wallis test (more than two groups).

CD4^+^ and CD8^+^ T cell responses specific to highly conserved epitopes, selected from these non-Spike antigens, were compared in unvaccinated asymptomatic individuals (those individuals who never developed any COVID-19 symptoms despite being infected with SARS-CoV-2) versus unvaccinated symptomatic COVID-19 patients (those patients who developed severe to fatal COVID-19 symptoms) (Fig. 2A). Unvaccinated HLA-DRB1*01:01^+^ and HLA-A*0201^+^ COVID-19 patients (*n* = 71) enrolled between January 2020 and December 2023, irrespective of the variant of concern with which they were infected, were divided into six groups based on the level of severity of their COVID-19 symptoms (increasing severity from 0 to 5), assessed at discharge (Fig. 2A). The clinical and demographic characteristics of this cohort of COVID-19 patients are detailed in our previous publication^41^. Fresh peripheral blood mononuclear cells (PBMCs) were isolated and then stimulated in vitro for 72 h using recently identified highly conserved 13 HLA-DR-restricted CD4^+^ or 16 HLA-A*0201-restricted CD8^+^ T cell peptide epitopes derived from the non-structural proteins (NSPs), the ORF7a//b, Membrane, Envelope, and Nucleoprotein, as detailed in “Methods.” The number of IFN-γ-producing CD4^+^ T and CD8^+^ T cells specific to epitopes from all 10 selected conserved antigens is shown in Fig. 2B. Specifically, 13 individual cross-reactive CD4^+^ T cell epitopes (Supplementary Fig. 2) and 16 individual cross-reactive CD8^+^ T cell epitopes (Supplementary Fig. 3) from the selected 10 highly conserved antigens were quantified in each of the six groups of COVID-19 patients using an ELISpot assay (i.e., number of IFN-γ-spot-forming T cells or “SFCs”). We then performed Pearson correlation analyses to assess the linear association between the magnitude of CD4^+^ and CD8^+^ T cell responses directed toward each conserved SARS-CoV-2 epitope and the severity of subsequent COVID-19 disease. A correlation is considered strong when the coefficient *R* value is between 0.7 and 1.

Overall, the highest frequencies of epitope-specific IFN-γ-producing CD4^+^ and CD8^+^ T cells (determined as mean SFCs >50 per 0.5 × 10^6^ PBMCs fixed as threshold) were detected in the unvaccinated COVID-19 patients who developed less severe disease (i.e., severity 0, 1, and 2, Fig. 2B, Supplementary Figs. 2 and 3). In contrast, the lowest frequencies of IFN-γ-producing CD4^+^ and CD8^+^ T cells were observed in unvaccinated COVID-19 patients who subsequently developed severe disease (severity scores 3 and 4; mean SFCs < 50) or died (severity score 5; mean SFCs <25). We found a strong positive linear correlation between the high magnitude of IFN-γ-producing CD4^+^ and CD8^+^ T cells specific to seven out of 10 common T cell antigens and the “natural protection” observed in unvaccinated asymptomatic COVID-19 patients (Fig. 2B, Supplementary Figs. 2 and 3). This positive correlation persisted regardless of whether CD4^+^ and CD8^+^ T cells targeted structural, non-structural, or accessory regulatory SARS-CoV-2 antigens.

Taken together, these results: (*i*) demonstrate an overall higher magnitude of CD4^+^ and CD8^+^ T cell responses specific to conserved non-Spike antigens present in unvaccinated asymptomatic COVID-19 patients irrespective of the SARS-CoV-2 variant of concern to which they were exposed; (*ii*) suggest a crucial role of these seven highly conserved structural, non-structural, and accessory regulatory T cell antigens, in protection from symptomatic and fatal Infections caused by multiple variants; and (*iii*) support these conserved non-Spike Coronavirus antigens as potential targets for a broad-spectrum CoV vaccine.

### Conserved SARS-CoV-2 NSP-2-, NSP-14- and Nucleoprotein-based mRNA/LNP vaccines confer protection against the highly pathogenic Delta variant (B.1.617.2)

We constructed methyl-pseudouridine-modified (m1Ψ) mRNAs encoding each of the 10 highly conserved T cell antigens (i.e., NSP-2, NSP-3, NSP-4, NSP-5-10, NSP-12, NSP-14, ORF7a/b, Membrane, Envelope, and Nucleoprotein), based on the Omicron sub-variant BA.2.75, using the CleanCap technology^42^. mRNA vaccines expressing the prefusion Spike protein stabilized by either two (Spike 2P) or six (Spike 6P) prolines were used as positive controls for B cell immunity^43,44^. The 12 mRNA vaccines were then encapsulated in lipid nanoparticles (LNPs)^45^. The mRNA/LNP platform was selected as the antigen-delivery technology because clinically proven Spike mRNA/LNP-based vaccines have been validated for safety and efficacy in humans and can be manufactured at a large scale to support rapid, global mass vaccination.

**Fig. 3:**
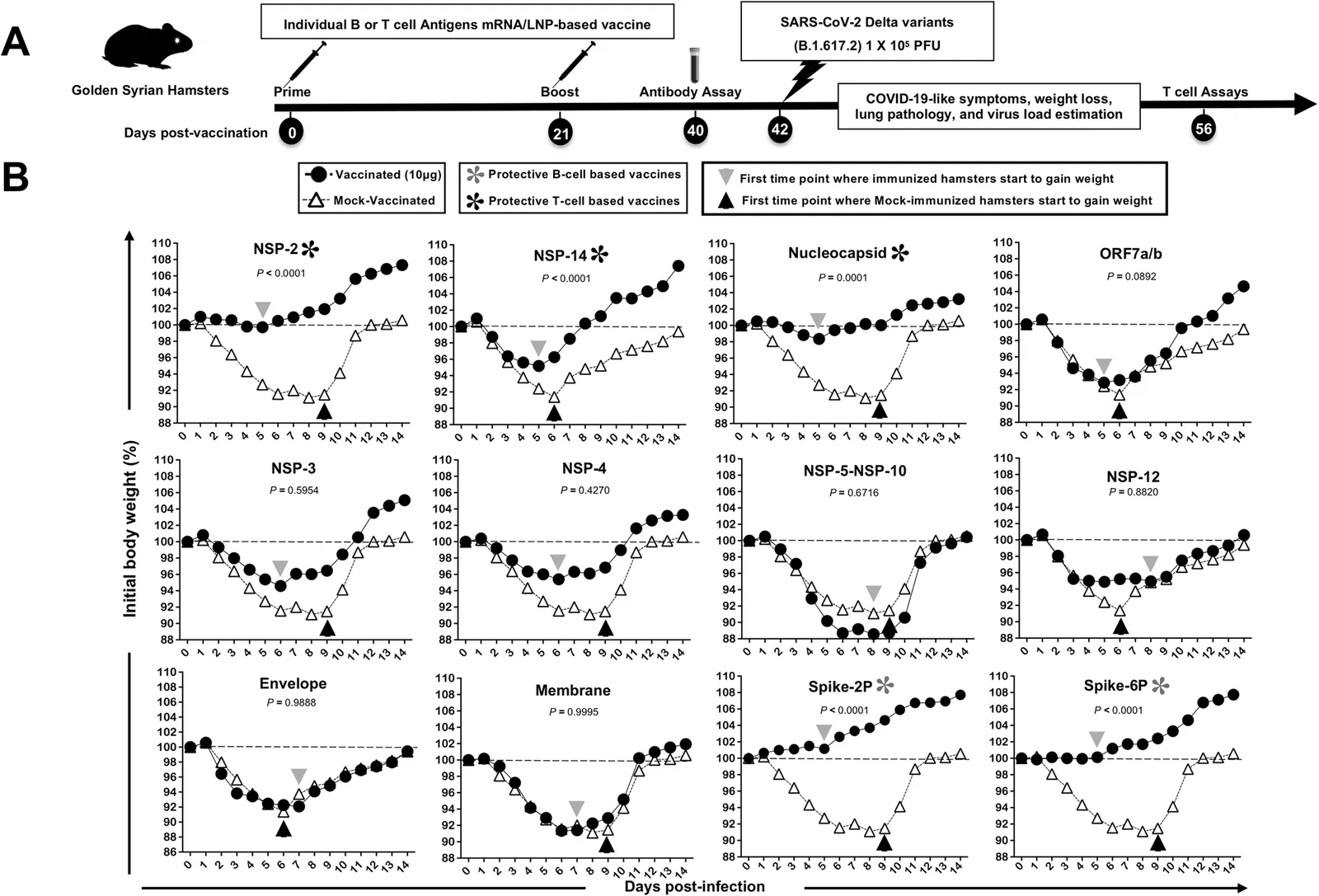
Screening of 10 highly conserved T cell antigens for protection against the highly pathogenic Delta variant (B.1.617.2) in golden Syrian hamsters. **A** Experimental plan to screen for vaccine efficacy. Male hamsters (*n* = 6 per group) were immunized intramuscularly on day 0 (prime) and day 21 (boost) with 10 mg/dose of the mRNA/LNP-based Coronavirus vaccines, each expressing 10 highly conserved non-Spike T-cell antigens. Hamsters that received phosphate-buffered saline alone were used as mock-immunized controls (*Saline*, *Mock*, *n* = 6). Three weeks after booster vaccination (day 42), vaccinated and mock-vaccinated hamsters were intranasally challenged (both nostrils) with 1 × 10^5^ pfu of SARS-CoV-2 highly pathogenic Delta variant (B.1.617.2). Weight losses were assessed for 14 days post-challenge. **B** Percent weight change for 14 days post-challenge normalized to the initial body weight on the day of infection in hamsters immunized with mRNA/LNP expressing NSP-2, NSP-3, NSP-4, NSP-5-10, NSP-12, NSP-14, ORF7a/b, Membrane, Envelope, Nucleoprotein, Spike 2P, and Spike 6P at 10 mg/dose. The dashed line indicates the 100% starting body weight. The arrowheads indicate the first day post-challenge when the weight loss is reversed in T cell- and Spike antigen-vaccinated (*gray arrowhead*) and mock-vaccinated (*black arrowhead*) hamsters. The immunogenicity and protective efficacy screening of the 12 antigens were performed in two phases. In the first phase, we screened for the immunogenicity and protective efficacy of NSP-2, NSP-3, NSP-4, NSP5-NSP10, Spike-2P, Spike-6P, Membrane, and Nucleoprotein antigens, as well as a Mock-vaccinated control. In the second phase, we screened for immunogenicity and protective efficacy of the remaining NSP-12, NSP-14, Envelope, and ORF7a/b antigens, together with a Mock-vaccinated control. The Mann–Whitney test (for two groups) was used for statistical analysis in both phases.

To screen for protective efficacy, each of the mRNA/LNP-based vaccines were delivered individually by the intramuscular route to outbred golden Syrian hamsters, then challenged with the highly pathogenic Delta variant (B.1.617.2) (Fig. 3A). Golden Syrian hamsters are naturally susceptible to SARS-CoV-2 infection, owing to the high degree of similarity between hamster ACE2 and human ACE2 (hACE2), and develop symptoms of COVID-19-like disease that closely mimic the COVID-19 pathogenesis in humans^46–50^. Male golden Syrian hamsters (*n* = 6 per group) were immunized intramuscularly on day 0 (prime) and day 21 (boost) with individual mRNA/LNP-based vaccines at a 10 μg/dose (Fig. 3A), based on previous similar mRNA-LNP vaccine studies in mice and hamsters^40,51^. Hamsters that received phosphate-buffered saline (PBS) alone were used as mock-immunized controls (*Saline*, *Mock*, *n* = 6). Power analysis indicated that five hamsters per group were sufficient to achieve a power > 80%. Three weeks after the second immunization, hamsters were challenged intranasally with the SARS-CoV-2 Delta variant (B.1.617.2) (1 × 10^5^ pfu per nostril). The challenge virus dose was determined by titration studies comparing 5 × 10^4^ pfu, 1 × 10^5^ pfu, and 5 × 10^5^ pfu; the middle dose of 1 × 10^5^ pfu was sufficient to produce substantial disease in hamsters (data not shown).

**Fig. 4:**
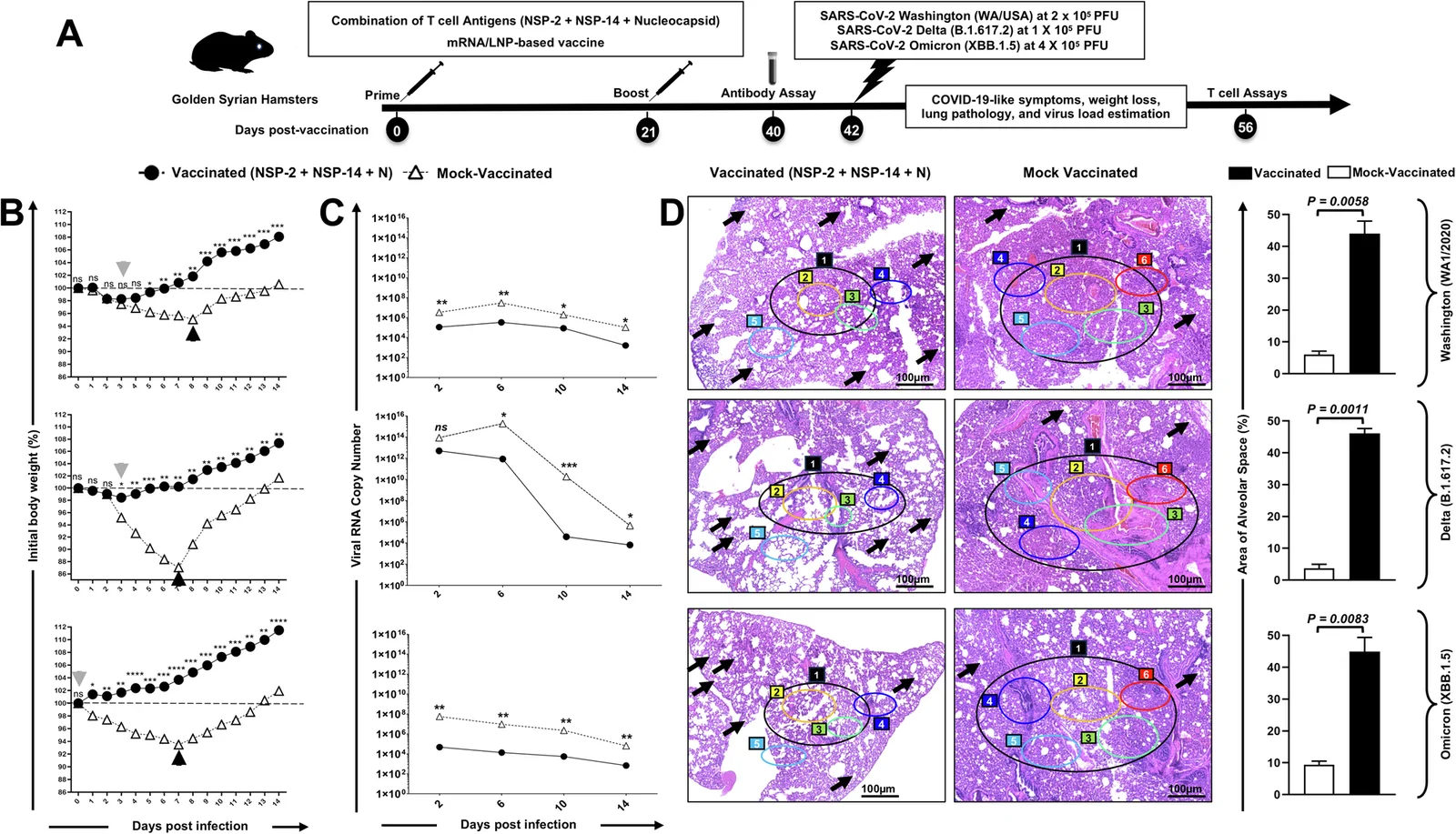
Protection against multiple SARS-CoV-2 variants induced by a combined NSP-2, NSP-14, and Nucleoprotein-based mRNA/LNP vaccine in the hamster model. **A** Experimental plan showing the timeline of the experiment. Three weeks after booster vaccination (day 42), vaccinated and mock-vaccinated hamsters were intranasally challenged (both nostrils) with 2 × 10^5^ pfu of the wild-type Washington variant (WA1/2020), 1 × 10^5^ pfu of the highly pathogenic Delta variant (B.1.617.2), or 2 × 10^5^ pfu of the highly transmissible Omicron sub-variant (XBB1.5). COVID-19 disease parameters, including **B** initial body percent weight loss, **C** viral RNA copy number, and **D** lung pathology, were assessed for 14 days post-challenge. **B** Percent weight change for 14 days post-challenge, normalized to the initial body weight on the day of infection for each variant and sub-variant. The dashed line indicates the 100% starting body weight. The arrowheads indicate the first day post-challenge when the weight loss is reversed in T cell- and Spike antigen (*gray arrowhead*) vaccinated and mock (*black arrowhead*) vaccinated hamsters. **C** Two-, 6-, 10-, 14-day post-infection (p.i.), viral loads were analyzed by comparing viral RNA copies in the hamster’s oropharyngeal swabs between mock and vaccine groups. **D** Representative H & E staining images of lung pathology at day 14 p.i. of SARS-CoV-2 infected hamsters, mock vaccinated or vaccinated with the combined NSP-2, NSP-14, and Nucleoprotein-based mRNA/LNP vaccine at 4× magnification. H&E-stained lung sections were evaluated for histopathologic evidence of SARS-CoV-2 Delta variant-induced lung injury and diffuse alveolar damage. On the H&E-stained lung slides, the following characteristics are numbered: 1 = diffuse alveolar damage (DAD), assessed as a composite pattern. DAD includes 2 = alveolar septal thickening, 3 = inflammatory cell infiltration, 4 = pneumocyte injury, 5 = intra-alveolar proteinaceous exudate, and 6 = hyaline membrane formation. Septal thickening was identified by alveolar wall widening and increased cellularity, with narrowing of the airspaces. Inflammatory infiltration was assessed by the presence of increased numbers of hematoxylin-positive inflammatory cells within septa, perivascular regions, or airspaces. Proteinaceous exudate was identified as amorphous eosinophilic material within alveolar or airway lumens. Hyaline membranes were identified as linear eosinophilic membrane-like deposits lining alveolar surfaces. Pneumocyte injury was assessed by epithelial attenuation, denudation, sloughing, or reactive pneumocyte morphology. Vaccinated hamsters (NSP-2 + NSP-14 + Nucleoprotein) exhibit a significantly reduced number of cytoplasmic vacuoles in pneumocytes (black arrows) compared to mock-vaccinated hamsters, indicating attenuation of lung injury following vaccination. Conversely, severe lung pathology in mock-vaccinated hamsters is characterized by a markedly reduced number of lung vacuoles (black arrows), consistent with extensive pneumocyte loss and diffuse alveolar damage. Lung pathology was quantified by determining the percentage area of alveolar space in lung sections from vaccinated and mock-vaccinated groups using ImageJ (Version 1.54p). Statistical comparisons were performed using the Mann–Whitney test (two groups) or the Kruskal–Wallis test (more than two groups); ns *P* > 0.05; *P < 0.05; **P < 0.01; ***P < 0.001; ****P < 0.0001.

Following virus challenge, mock-vaccinated hamsters progressively lost up to 10% of their body weight within the first week after infection, before gradually returning to their original weight by approximately day 12. Hamsters that received the mRNA/LNP vaccine expressing Spike 2P or Spike 6P were protected against weight loss following virus challenge (*P* ≤ 0.001, Fig. 3B). Conserved T cell antigens mRNA/LNP-based vaccines, NSP-2, NSP-14, and Nucleoprotein also prevented weight loss (*P* < 0.05, Fig. 3B) with NSP-2 was the most protective (only 2% body weight loss), followed by Nucleoprotein (4% body weight loss) and NSP-14 (6% body weight loss). Faster recovery was also observed in hamsters vaccinated with the Spike and T cell antigens, as measured by reversal of their weight loss as early as 4–5 days after challenge (*Gray arrows*, Fig. 3B), versus 6–9 days for the mock-vaccinated hamsters (*Black arrows*, Fig. 3B) (see also Fig. 4A). NSP-3 was another conserved T cell antigen that showed a trend toward protection against weight loss, but along with the remaining 6 T cell antigens (i.e., NSP-4, NSP-5-10, NSP-12, Membrane, and Envelope) did not produce any significant protection against weight loss (*P* > 0.05, Fig. 3B). Similar protection data as measured by SARS-CoV-2 viral RNA copy number in hamsters on Days 2, 6, 10, and 14 post-challenge, were observed with substantial reductions in virus titer (up to 4 logs) in animals immunized with NSP-2, NSP-14, Nucleoprotein and the Spike vaccines (Supplementary Fig. 5B). NSP-3 and NSP-4 appeared to confer protection early after challenge (days 2 and 6) but not later. No significant protection was observed with any of the other T-cell antigens. These protection data correlated with the level of lung pathology, where reduced COVID-19 pathology was observed in hamsters immunized with NSP-2, NSP-14, Nucleoprotein, and the Spike vaccines compared to those vaccinated with ORF7a/b, NSP-3, NSP-4, NSP-5-NSP-10, NSP-12, Envelope, and Membrane (Supplementary Fig. 6B).

The NSP-2, NSP-14, and Nucleoprotein were specifically selected for further analyses based on (1) better conservation of the sequences in multiple variants and sub-variants; and (2) better protection these antigens induced, compared to the remaining 10 T cell antigens, as evaluated using multiple criteria, including protection from weight loss, reduction of virus titers, and lung immunopathology (Fig. 3, Supplementary Figs. 5 and 6).

### A NSP-2-, NSP-14-, and Nucleoprotein-based mRNA/LNP combination vaccine conferred robust and broad protection against multiple SARS-CoV-2 variants and sub-variants of concern

We next determined the protective efficacy of a combination mRNA/LNP vaccine consisting of the NSP-2, NSP-14 and Nucleoprotein T cell antigens (Fig. 4A), against VOCs with various characteristics, including an ancestral wild-type Washington variant (WA1/2020), the highly pathogenic Delta variant (B.1.617.2), and the heavily Spike-mutated and highly transmissible Omicron sub-variant (XBB.1.5). Male golden Syrian hamsters were immunized intramuscularly on day 0 and day 21 with the combination vaccine at a 1 μg/dose for each component (3 μg/dose total) (*n* = 6 per group) or mock-immunized (*n* = 6 per group). Three weeks after the second immunization, animals were divided into groups of 5 hamsters each and challenged intranasally, in both nostrils, with 2 × 10^5^ pfu of the wild-type Washington variant (WA1/2020) (*n* = 6 per group), 1 × 10^5^ pfu of Delta variant (B.1.617.2) (*n* = 6 per group) or 2 × 10^5^ pfu of Omicron sub-variant (XBB1.5) (*n* = 6 per group), based on virus titration studies to determine an optimal effective dose in hamsters (data not shown).

Vaccination with the NSP-2, NSP-14, and Nucleoprotein-based combination vaccine substantially prevented weight loss (Fig. 4B), reduced virus titer by several logs (Fig. 4C), and significantly reduced lung pathology (Fig. 4D) following challenge with wild-type Washington variant (WA1/2020), Delta variant (B.1.617.2), and Omicron sub-variant (XBB1.5). Vaccinated hamsters exhibit a significantly reduced number of cytoplasmic vacuoles in pneumocytes (black arrows) compared to mock-vaccinated hamsters, indicating attenuation of lung injury following vaccination. Conversely, severe lung pathology in mock-vaccinated hamsters is characterized by a markedly reduced number of lung vacuoles (black arrows), consistent with extensive pneumocyte injury and diffuse alveolar damage (DAD) (Fig. 4D, *Left panel*). Of particular interest, 6 out of 6 hamsters that received the combination vaccine and were challenged with the heavily Spike-mutated and transmissible Omicron sub-variant (XBB.1.5) did not lose any weight and had only marginal weight loss after the Washington and Delta virus challenge, versus up to 13% weight loss in the mock-vaccinated animals. In addition, animals vaccinated with the combination vaccine recovered more quickly after challenge (0–3 days) than mock-vaccinated animals (7 days). Fourteen days post-challenge, lung tissues were collected and fixed, and 8-μm sections were cut from hamsters and stained with hematoxylin and eosin (H&E). The lungs of hamsters that received the combined NSP-2, NSP-14, and Nucleoprotein-based mRNA/LNP vaccine demonstrated regular bronchial, bronchiolar, and alveolar architecture (Fig. 4D). A reduced degree of inflammation (black arrows) was observed in the vaccinated group of hamsters in comparison to mock-vaccinated hamsters. In contrast, the lungs of mock-immunized hamsters showed acute bronchiolitis with adjacent marked interstitial pneumonia. Taken together, these results demonstrate that the combination of NSP-2, NSP-14, and Nucleoprotein provides rapid, robust, and broad protection against infection and disease caused by multiple SARS-CoV-2 variants and subvariants of concern.

### A Spike, NSP-2, NSP-14, and Nucleoprotein-based combination mRNA/LNP vaccine induced stronger, faster, and broader protection against multiple variants and sub-variants compared to the Spike-alone-based mRNA/LNP vaccine

**Fig. 5:**
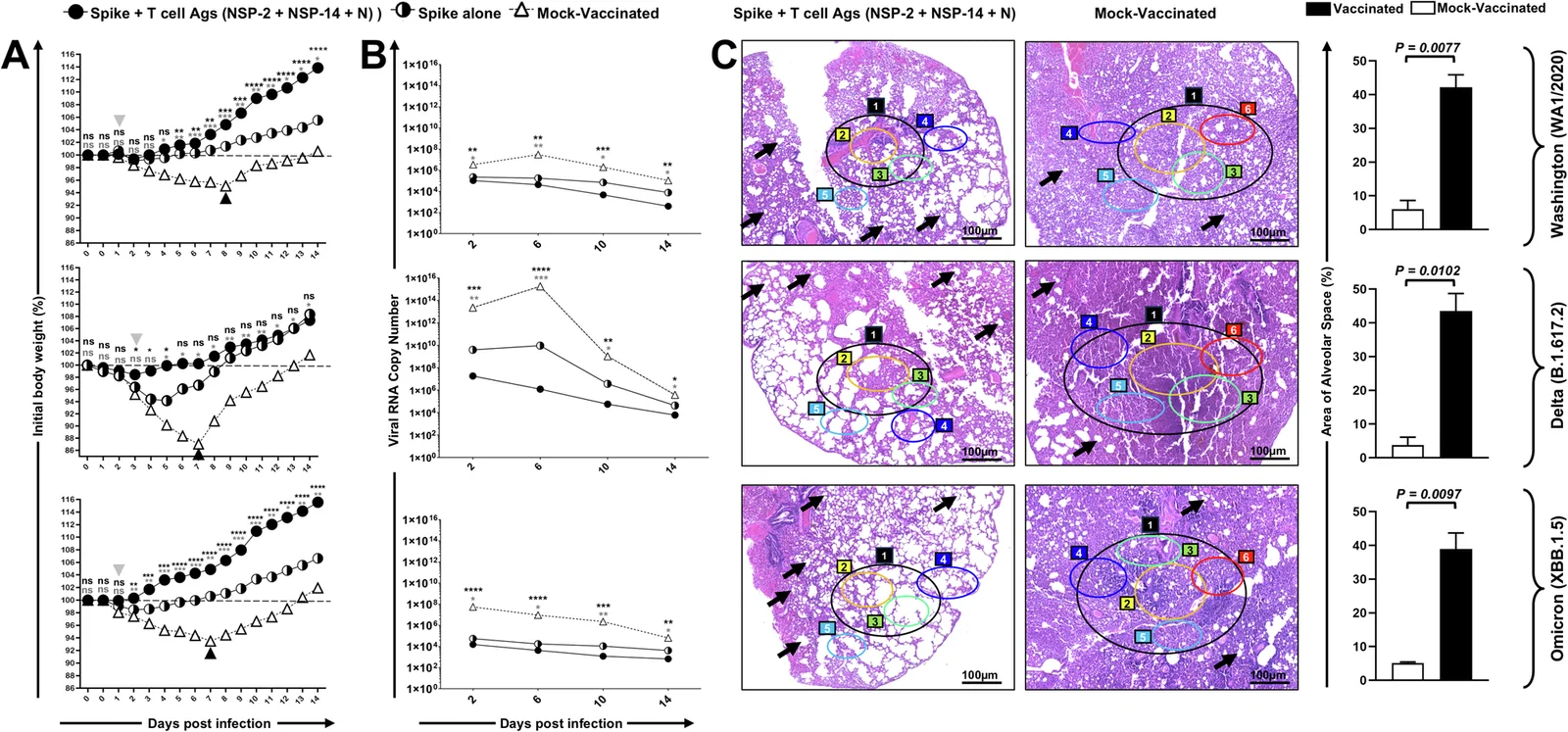
Superior protection induced by combined Spike, NSP-2, NSP-14, and Nucleoprotein-based mRNA/LNP vaccine against the highly pathogenic Delta variant (B.1.617.2). **A** Percent weight change for 14 days post-challenge normalized to the initial body weight on the day of infection with the highly pathogenic Delta variant (B.1.617.2). The dashed line indicates the 100% starting body weight. **B** Virus titers were analyzed at 2-, 6-, 10-, and 14-day post-infection (p.i.) to evaluate vaccine-induced protection against virus replication by comparing viral RNA copies in the hamsters’ throats and lungs between the mock and vaccine groups. Viral RNA copies were quantified by RT-PCR and expressed as log_10_ copies per milligram of throat or lung tissue. The graphs compare viral titers in the lungs of vaccinated and mock-vaccinated hamsters. **C** Representative H & E staining images of lung pathology at day 14 p.i. of SARS-CoV-2 infected hamsters, mock vaccinated or vaccinated with the combined Spike, NSP-2, NSP-14, and Nucleoprotein-based mRNA/LNP vaccines at 4x magnifications. Significantly reduced number of lung vacuoles (black arrows) in the lung tissues is found in the hamsters non-vaccinated with combined T cell antigens (NSP-2 + NSP-14 + Nucleoprotein) along with Spike antigen, in comparison to the vaccinated hamsters. Severe lung pathology in Mock-vaccinated hamsters is characterized by a reduced number of lung vacuoles (black arrows), along with prominent histopathological features. On the H&E-stained lung slides, the following histopathological characteristics are numbered: 1 = diffuse alveolar damage (DAD), assessed as a composite pattern. DAD includes 2 = alveolar septal thickening, 3 = inflammatory cell infiltration, 4 = pneumocyte injury, 5 = intra-alveolar proteinaceous exudate, and 6 = hyaline membrane formation. These findings collectively indicate extensive alveolar damage and disruption of normal pulmonary architecture in unprotected animals. To preserve the integrity of the lung tissue and ensure accurate morphometric assessment, lungs were gently inflated with fixative before processing, a critical step to prevent pre-collapse of lung tissue and avoid artificial compression of airspaces that can occur in portions of the lung when inflation is inadequate. Lung pathology is quantified by means of the percentage area of alveolar space in the lungs of vaccinated and mock-vaccinated groups of hamsters. The graphed values and bars represent the standard deviation (SD) between the two experimental groups. The Mann–Whitney test (for two groups) or the Kruskal–Wallis test (for more than two groups) was used for statistical analysis. ns *P* > 0.05, *P < 0.05, **P < 0.01, ***P < 0.001, ****P < 0.0001. The upper and lower asterisks denote comparisons between the combination vaccine and the mock-vaccinated and Spike-alone vaccines, respectively.

We next investigated whether the inclusion of NSP-2, NSP-14, and Nucleoprotein, together with Spike, would improve the protective efficacy of a Spike-alone vaccine, which represents the current standard of care. For this experiment, we selected the prefusion Spike protein stabilized by two prolines (Spike-2P) to align with the clinically proven Spike mRNA/LNP-based vaccines. We first demonstrated the functionality of the individual mRNA components by measuring expression of the four proteins after in vitro mRNA transfection of human epithelial HEK293T cells (*white arrows*, Supplementary Fig. 4). Co-transfection of the four mRNA vaccines did not result in apparent competition, as all four antigens were expressed at comparable levels in vitro (data not shown). The efficacy of the Spike, NSP-2, NSP-14, and Nucleoprotein combination vaccine was compared to the Spike-alone vaccine at a dose of 1 μg/component against multiple variants (Fig. 5A). Hamsters that received the combination vaccine were substantially better protected from weight loss compared to those that received the Spike-only vaccine after challenge with the wild-type Washington variant (USA-WA1/2020) (Fig. 5A, *Top Panel*), highly pathogenic Delta variant (B.1.617.2) (Fig. 5A, *Middle Panel*), or highly transmissible Omicron sub-variant (XBB.1.5) (Fig. 5A, *Bottom Panel*). This superior protection was evidenced by lower weight loss and faster recovery (0–2 days versus 4–6 days). As expected, the mock-vaccinated hamsters rapidly lost weight and did not regain weight until 7–8 days after the challenge. The virus titers determined on days 2, 6, 10, and 14 post-challenge confirmed the significant reduction in viral burden conferred by the combination vaccine versus mock-vaccinated controls (up to 8 logs) and the Spike-alone vaccine (up to 2 logs) following challenge (Fig. 5B). Histopathological analysis revealed that the lungs of hamsters receiving the combination vaccine exhibited standard bronchial, bronchiolar, and alveolar architecture (Fig. 5C, *Left Panel*). In contrast, considerable pathological changes, including bronchitis and interstitial pneumonia, are evident in the lungs of mock-immunized hamsters on 14 days post-challenge (Fig. 5C, *Right Panel*). Specifically, a significantly reduced number of lung vacuoles (black arrows), along with prominent histopathological features, including DAD, evaluation of alveolar septal thickening, inflammatory cell infiltration, proteinaceous exudate, hyaline membrane formation, and pneumocyte injury, are found in mock-vaccinated hamsters in comparison to the vaccinated hamsters (Fig. 5C, *Left Panel*). Together, the results (*i*) demonstrate that the combined Spike, NSP-2, NSP-14, and Nucleoprotein-based mRNA/LNP vaccine induced stronger and broader protection against multiple variants and sub-variants versus the Spike-only vaccine; and (*ii*) suggest that T cell responses directed toward the conserved T cell antigens may have provided broader protection in the face of immune escape by the heavily Spike-mutated variants, compared to the Spike-alone-based mRNA/LNP vaccine.

### Robust and long-lasting protection against the highly pathogenic SARS-CoV-2 Delta (B.1.617.2) and recently circulating SARS-CoV-2 Omicron KP.3 sub-variant induced by vaccination

**Fig. 6:**
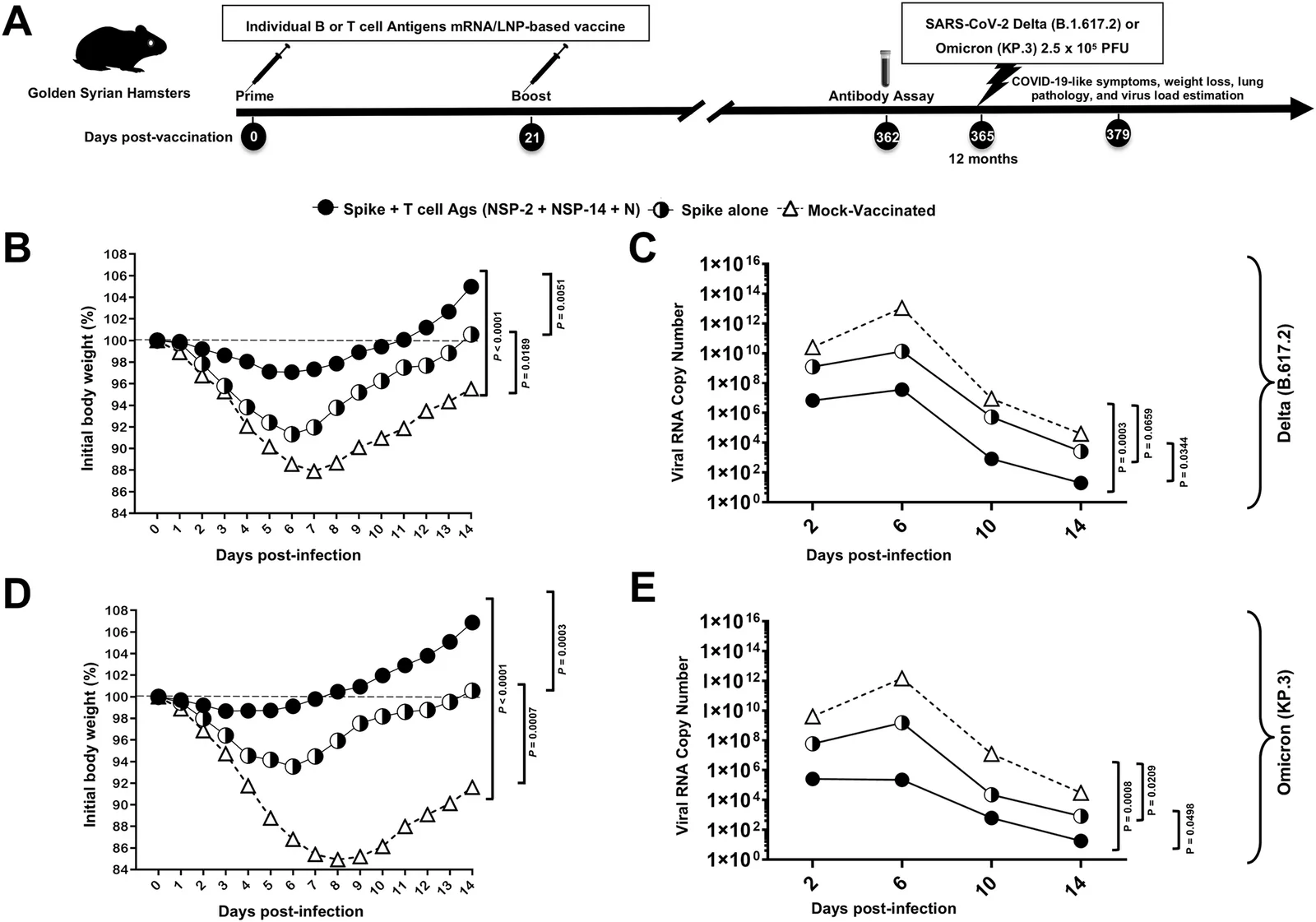
Robust protection induced by combined NSP-2, NSP-14, and Nucleoprotein-based mRNA/LNP vaccine after 1 year post vaccination against currently circulating SARS-CoV-2 Omicron KP.3 and highly pathogenic SARS-CoV-2 Delta variant in the hamster model. **A** Experimental design and timeline to study the vaccine efficacy in golden Syrian hamsters vaccinated with a combination of Spike, NSP-2, NSP-14, and Nucleoprotein-based and Spike alone-based mRNA/LNP vaccine. Hamsters were immunized intramuscularly twice on day 0 (prime) and day 21 (boost) with the combined Spike, NSP-2, NSP-14, and Nucleoprotein-based (*n* = 6 per group), Spike-alone (*n* = 6 per group), or mock-vaccinated (*Mock*, *n* = 6 per group). One year (377 days) after booster vaccination, vaccinated and mock-vaccinated hamsters were intranasally challenged (in both nostrils) with 2.5 × 10^5^ pfu of the current circulating Omicron KP.3 variant or the highly pathogenic Delta variant (B.1.617.2). COVID-19-like symptoms, lung pathology, weight loss, and virus load were assessed for 14 days post-challenge. For the SARS-CoV-2 Delta variant-specific challenge (**B**), the percent weight change over 14 days post-challenge, normalized to the initial body weight on the day of infection, is shown. The dashed line indicates the 100% starting body weight. **C** Viral RNA Copy number shown at 2-, 6-, 10-, 14-days post-infection (p.i.) for Delta variant specific challenge. For the SARS-CoV-2 Omicron KP.3 variant-specific challenge, **D** percent weight change for 14 days post-challenge, normalized to the initial body weight on the day of infection, is shown. The dashed line indicates the 100% starting body weight. **E** Omicron KP.3 variant-specific viral RNA Copy number shown at 2-, 6-, 10-, 14-days post-infection (p.i.). The Mann–Whitney test (for two groups) or the Kruskal–Wallis test (for more than two groups) was used for statistical analysis. ns *P* > 0.05, *P < 0.05, **P < 0.01, ***P < 0.001, ****P < 0.0001.

Next, we have evaluated the long-term protection of the combined Spike-, NSP-2-, NSP-14-, and Nucleoprotein-based vaccine versus Spike-alone delivered at a dose of 1 μg of mRNA/component. At 1 year (i.e., 365 days) following vaccination, separate groups of hamsters were challenged with either the highly pathogenic SARS-CoV-2 Delta (B.1.617.2) or the recently circulating variant SARS-CoV-2 Omicron (KP.3) at 2.5 × 10^5^ pfu (Fig. 6A). Strong protection against both virus strains, as measured by weight loss (Fig. 6B, D) and virus titration (Fig. 6C, E), was induced by the combination vaccine. In contrast, the Spike alone-based mRNA/LNP vaccine induced more modest protection. Consistently robust and superior protection was observed at 3-, 6-, 9-, and 12-month post-vaccination against challenge with SARS-CoV-2 Delta (B.1.617.2) (see Supplementary Fig. 7 and data not shown).

### Enriched polyfunctional lung-resident antigen-specific CD4^+^ and CD8^+^ T cells and neutralizing antibodies are induced by the combined Spike, NSP-2, NSP-14, and Nucleoprotein-based combination mRNA/LNP vaccine

**Fig. 7:**
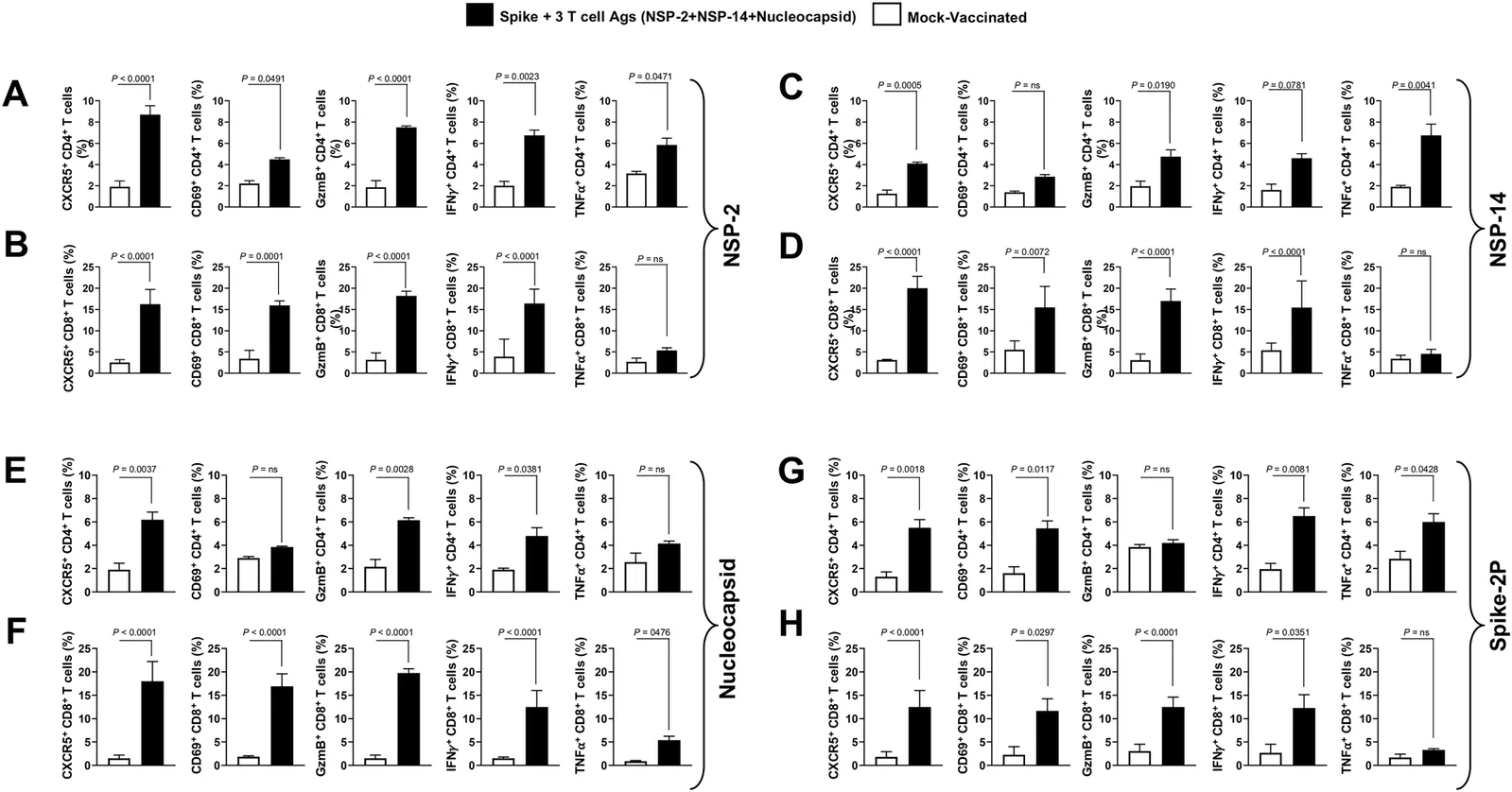
Lung-resident antigen-specific functional CD4^+^ and CD8^+^ T cells induced by the combined NSP-2, NSP-14, and Nucleoprotein-based mRNA/LNP vaccines in the hamsters after 1 year post vaccination. The panel shows average frequencies of functional CD4^+^ and CD8^+^ T cells after 1 year post vaccination in the lungs of hamsters vaccinated with the combined NSP-2, NSP-14, Nucleoprotein, and Spike-based mRNA/LNP vaccines. The graphs depict the levels of **A**, **B** NSP-2-specific, **C**, **D** NSP-14-specific, **E**, **F** Nucleoprotein- and **G**, **H** Spike-specific CD4^+^ and CD8^+^ cells present in the lungs at 14 days after challenge with the Omicron KP.3 variant in vaccinated (black bars) and mock-vaccinated (open bars) animals. The graphed values and bars represent the standard deviation (SD) between the two experimental groups. The Mann–Whitney test (for two groups) was used to analyze the data. Results were considered statistically significant at *P* < 0.05.

Finally, we determined whether the observed rapid and broad protection against SARS-CoV-2 disease and infection in hamsters vaccinated with the combined Spike-, NSP-2-, NSP-14-, and Nucleoprotein-based mRNA/LNP vaccine was associated with antigen-specific lung-resident T cell responses and neutralizing antibodies. Lungs from vaccinated and mock-vaccinated hamsters were collected 2 weeks after SARS-CoV-2 challenge, and cell suspensions were stimulated with pools of 15-mer peptides overlapping NSP-2, NSP-14, Nucleoprotein, or Spike. The frequency and function of antigen-specific CD8^+^ and CD4^+^ T cells were compared in vaccinated protected hamsters versus mock-vaccinated unprotected hamsters (Fig. 7). The data showed that the combination vaccine elicited robust NSP-2- (Fig. 7A, B), NSP-14- (Fig. 7C, D), Nucleoprotein-(Fig. 7E, F), and Spike-specific (Fig. 7G, H), CD4^+^ (Fig. 7A, C, E, G), and CD8^+^ T (Fig. 7B, D, F, H) cell responses, as measured by IFN-γ, Granzyme B, CD69, TNFα, and CXCR5 expression, compared to the mock-vaccinated animals.

Since the combined Spike-, NSP-2-, NSP-14-, and Nucleoprotein-based mRNA/LNP vaccine induced strong NSP-2-, NSP-14-, and Nucleoprotein-specific CXCR5^+^CD4^+^ T_FH_ cells, we determined whether the combination vaccine would induce better Spike-specific antibody responses. Serum samples were collected after vaccination and before the viral challenge for analysis by ELISA and neutralization assays. Higher titers of Spike-specific IgG-specific antibodies were detected in five out of five hamsters that received the combination vaccine compared to hamsters that received the Spike-alone vaccine (Supplementary Fig. 7D). Similarly, higher levels of neutralizing antibodies were elicited by the combination vaccine compared to the Spike-alone-based mRNA/LNP vaccine (Table 1). Modest but consistently higher neutralizing antibodies (up to 4-fold) were observed against the Delta, Washington, and Omicron variants, suggesting that the combination vaccine may have elicited additional T-cell helper responses, resulting in a more potent antibody response.

**Table 1:** Neutralizing antibodies at 3-, 6-, 9-, and 12-months post immunization

SARS-CoV-2 variant *(Time indicates months post-vaccination)*	Spike EC_50_ values	Spike + 3 T cell Ags (NSP-2 + NSP-14 + Nucleoprotein) EC_50_ values	EC_50_ fold increase	*P* values
**SARS-CoV-2 Delta (B.617.2)**				
Delta (B.617.2)—3 months	5000	10,971	1.19	0.024
Delta (B.617.2)—6 months	846	2788	2.24	0.036
Delta (B.617.2)—9 months	2025	2881	0.42	0.029
Delta (B.617.2)—12 months	846.8	1164	0.37	0.048
**SARS-CoV-2 Washington (WA/USA)**				
Washington (WA/USA)—3 months	8365	24,201	1.88	0.032
Washington (WA/USA)—6 months	1559	3564	1.86	0.041
Washington (WA/USA)—9 months	959	3389	2.53	0.039
Washington (WA/USA)—12 months	1873	2315	0.23	0.028
**SARS-CoV-2 Omicron (BA.2)**				
Omicron (BA.2)—3 months	5522	13,259	1.40	0.016
Omicron (BA.2)—6 months	803	2943	2.66	0.028
Omicron (BA.2)—9 months	335	728	1.17	0.041
Omicron (BA.2)—12 months	1485	2071	0.39	0.046

Neutralization assays were performed against the SARS-CoV-2 Washington, Delta, and Omicron variants at 3-, 6-, 9-, and 12-month post-vaccination in animals immunized with Spike alone or the combination vaccine containing Spike and T cell antigens. Shown are EC_50_ values, fold increases compared to Spike alone, and *P* values, as measured using the Student’s *t*-test.

### CD4^+^ and CD8^+^ T cells depletion led to the abrogation of mRNA-LNP vaccines’ protection against SARS-CoV-2 Delta (B.1.617.2) variant in hACE2 mice

The effects of CD4^+^ and CD8^+^ T cell depletion on percent body weight loss and viral titration against intranasal infection with SARS-CoV-2 Delta (B.1.617.2) variant were analyzed in mRNA-LNP immunized hACE2 mice. The immunized hACE2 mice were subjected to CD4^+^ and/or CD8^+^ T cell depletion, followed by challenge with 1 × 10^5^ pfu of SARS-CoV-2 Delta (B.1.617.2) variant. The mice were followed up for 14 days post-intranasal infection, and their weight and survival were measured daily. CD8^+^ and CD4^+^ T cells of the immunized mice were depleted by intraperitoneal injection (two times) of anti-CD4 and/or anti-CD8α mAbs. Although all mice challenged with SARS-CoV-2 start losing weight 2 days post-infection, only Immunized mice with mRNA-LNP encoding a combination of T cell Antigens (NSP-2 + NSP-14 + Nucleoprotein) start regaining weight by days 5 and 6. Mice subjected to no T cell depletion showed no significant weight loss and started regaining weight from Day 5 onwards in the experiment (Supplementary Fig. 9A). Further, we have found that the mice subjected to CD8^+^ T cell depletion had small impact on the loss of body weight in contrast to CD4^+^ T-cell depletion and both CD4+ and CD8+ T-cells depletion (Supplementary Fig. 9A). This suggested that T cells play an important role in preventing weight loss in mice immunized with Combination of the T cell Antigens (NSP-2 + NSP-14 + Nucleoprotein) and challenged with SARS-CoV-2. The percent body weight loss data were complemented with the viral titration data that showed a similar pattern (Supplementary Fig. 9B).

## Discussion

The Coronavirus disease 2019 (COVID-19) pandemic has created one of the most significant global health crises in nearly a century^1–6^. The number of confirmed SARS-CoV-2 cases has reached over 777 million, and COVID-19 has caused almost 7.2 million deaths^1,5,6^. As of July 2025, the world is entering its sixth and half year of a persistent COVID-19 pandemic, fueled by the continuous emergence of heavily Spike-mutated and highly contagious SARS-CoV-2 variants and sub-variants that: (*i*) escape immunity induced by the current clinically proven Spike-alone-based vaccines; (*ii*) disrupt the efficacy of the COVID-19 booster paradigm^7,9,10,52–54^; and (*iii*) outpace the development of variant-adapted Spike-alone vaccines^1,4–6,23^. This bleak outlook of a prolonged COVID-19 pandemic underscores the urgent need for developing a next-generation, broad-spectrum CoV vaccine capable of conferring strong cross-variant and cross-strain protective immunity to prevent immune evasion and breakthrough infections, thereby ending the COVID-19 pandemic^4^.

In the present preclinical vaccine study, employing in silico, in vitro, and in vivo approaches, we screened the SARS-CoV-2 genome for conserved viral antigens that could serve as targets for protective T-cell-mediated immunity. We identified three such antigens: NSP-2, NSP-14, and Nucleoprotein. Here, we demonstrate that a combination mRNA/LNP vaccine, consisting of Spike, NSP-2, NSP-14, and Nucleoprotein, induces superior, durable, broad, and cross-protective immunity compared to a Spike-only vaccine, representative of the current standard of care, against several highly contagious and heavily Spike-mutated SARS-CoV-2 variants and subvariants. The benefit of including NSP-2, NSP-14, and Nucleoprotein was also demonstrated by using a combination vaccine containing the licensed vaccine Spikevax^TM^ as the Spike component (*not shown*). These three T cell antigens are: (*i*) Expressed by the early transcribed virus RTC region; (*ii*) Preferentially targeted by human cross-reactive memory CD4^+^ and CD8^+^ T cells associated with protection of asymptomatic COVID-19 patients (i.e., unvaccinated individuals who never develop any COVID-19 symptoms despite being infected with SARS-CoV-2); and (*iii*) selectively targeted by lung-resident enriched memory CD4^+^ and CD8^+^ T cells from SARS-CoV-2 exposed seronegative individuals who were able to rapidly abort the virus replication (i.e., “SARS-CoV-2 aborters”)^33–36^. Hamsters that received the combination mRNA/LNP vaccine displayed lower virus load, improved lung pathology, and protection from weight loss caused by various VOCs, including an ancestral wild-type Washington variant (WA1/2020), the highly pathogenic Delta variant (B.1.617.2), the heavily Spike-mutated Omicron sub-variants (B.1.1.529 and XBB1.5), as well as the recently circulating Omicron KP.3 variant.

We used stabilized SARS-CoV-2 Spike protein, containing either two prolines (Spike 2P) or six prolines (Spike 6P/HexaPro) substitution. This has enhanced the prefusion conformation required for higher protein expression and improved immunogenicity^55^. Spike 2P uses two proline substitutions (positions K986P and V987P) to stabilize the Spike’s prefusion structure; this design underlies the Pfizer, Moderna, and J&J COVID-19 vaccines^56^. Spike 6P (HexaPro) introduces four additional prolines (F817P, A892P, A899P, A942P), thereby further stabilizing the Spike protein, enhancing expression yields, and improving resistance to unfolding/denaturation^44^. Both methods lock the SARS-CoV-2 Spike in its antigenic prefusion structure; however, the six-proline variant (Spike 6P/HexaPro) appears more stable and immunogenic. mRNA vaccines expressing Spike 2P or Spike 6P/HexaPro were used as positive controls for B-cell immunity^43,44^. The 12 mRNA vaccines were then encapsulated in lipid nanoparticles (LNPs)^45^. The combination vaccine elicited high frequencies of NSP-2-, NSP-14-, and Nucleoprotein-specific follicular CXCR5^+^CD4^+^ T cells that produce IFN-γ and TNF-α compared with mock-vaccinated animals. The combination of three T cell antigens and Spike induced better neutralizing antibodies compared to Spike alone, likely because each of the three T cell antigens induced a high frequency of functional follicular CXCR5^+^CD4^+^ T cells, which in turn helps B-cell maturation and production of Spike-specific neutralizing antibodies. The potent and broad cross-protection induced by the combined mRNA/LNP vaccine was associated with enhanced Spike-specific IgG and neutralizing antibodies, as well as enriched lung-resident NSP-2-, NSP-14-, Nucleoprotein-, and Spike-specific CD4^+^ and CD8^+^ T follicular helper (T_FH_) cells, cytotoxic T cells (_CTL_), and effector T cells (T_EFF_). The critical role of CD4^+^ and CD8^+^ T-cells in the protection afforded by the combination vaccine is further supported by data showing that T-cell depletion abrogates protection in mice (Supplementary Fig. 9). These preclinical findings are consistent with the above reference data from humans and suggest that an alternative broad-spectrum CoV vaccine may be capable of: (*i*) disrupting the current COVID-19 booster paradigm; (*ii*) outpacing the variant-adapted COVID-19 vaccines; (*iii*) ending the ongoing COVID-19 pandemic, and (iv) preventing future CoV outbreaks.

The efficacy of first-generation Spike-alone-based COVID-19 vaccines is threatened by the emergence of numerous immune-evasive SARS-CoV-2 variants and subvariants that can evade protective neutralizing antibody responses^1,4–6,23^. While the Wuhan strain (Hu1) is the ancestral variant of SARS-CoV-2 that emerged in late 2019 in China, Alpha (B.1.1.7), Beta (B.1.351), and Gamma (B.1.1.28) VOCs subsequently appeared in the United Kingdom, South Africa, and Brazil, respectively, between 2020 and 2021^11^. The most pathogenic Delta variant (B. 1.617.2) was identified in India in mid-2021, where it led to a deadly wave of infections^11^. The highly Spike-mutated Omicron variants and subvariants (i.e., B.1.1.529, XBB1.5, BA.2.86, JN.1, KP.3, NB.1.8.1, and LP.8.1) emerged between 2021 and 2025 and are less pathogenic but more immune-evasive^9,10,31^. The waning immunity induced by Spike-alone vaccines, as well as the antigenic drift of SARS-CoV-2 variants, has diminished vaccine efficacy against many recent highly mutated Spike VOCs^4,31,57^. Emerging SARS-CoV-2 variants, particularly the Omicron lineages, which exhibit frequent mutations in the Spike protein, evade immunity induced by vaccination or natural infection^58,59^. Thus, first-generation Spike-based COVID-19 vaccines must be regularly updated to better match emerging variants. Despite the recent introduction of updated vaccines, breakthrough infections with the highly transmissible and highly Spike-mutated Omicron subvariants XBB1.5, EG.5, HV.1, BA.2.86, JN.1, KP.3, NB.1.8.1, and LPLP.8.1 are contributing to a prolonged COVID-19 pandemic^9,10,53^.

This preclinical study comprehensively characterized the safety, immunogenicity, and protective efficacy of SARS-CoV-2-derived T-cell antigens identified through a genome-wide screen, delivered as mRNA/LNP-based vaccine candidates. We identified five highly conserved regions in the SARS-CoV-2 single-stranded RNA genome that encode for three structural (Membrane, Envelope, and Nucleoprotein), 11 non-structural (NSP-2, NSP-3, NSP-4, NSP-5-10, NSP-12, NSP-14), and one accessory protein encoded by the open-reading frame, ORF7a/b^38^. Among these conserved viral proteins, we observed that the early-transcribed non-structural proteins, including NSP-2, NSP-7, NSP-12, NSP-13, and NSP-14, from the RTC region, as well as the structural Nucleoprotein, were selectively targeted by (*i*) peripheral blood cross-reactive memory CD4^+^ and CD8^+^ T cells from asymptomatic COVID-19 patients. This is in agreement with our and others reports that detected high frequencies of cross-reactive functional CD4^+^ and CD8^+^ T cells directed toward specific sets of conserved SARS-CoV-2 non-Spike antigens, including NSP-2, NSP-7, NSP-12, NS-13, NSP-14 and Nucleoprotein in unvaccinated asymptomatic COVID-19 patients^5,24–30^; and (*ii*) by lung-resident cross-reactive memory CD4^+^ and CD8^+^ T cells associated with rapid clearance of infection in so-called “SARS-CoV-2 aborters”^33–37^. The vigorous and enriched cross-reactive RTC-specific CD4^+^ and CD8^+^ T-cells mounted by “SARS-CoV-2 aborters” spontaneously “abort” virus infection so rapidly that they never presented detectable SARS-CoV-2 infection, despite constant exposure to the virus^33–36^. Similarly, we found the NSP-2, NSP-14, and Nucleoprotein, which are incorporated in our combination mRNA/LNP vaccine, were also targeted by enriched lung-resident antigen-specific T follicular helper (T_FH_) cells, cytotoxic T cells (T_CYT_), and effector T cells (T_EFF_) associated with the rapid clearance of the virus from the lungs of protected hamsters^60,61^. These findings suggest that early-expressed conserved antigens in the RTC region, selectively recognized by CD4^+^ and CD8^+^ T cells from asymptomatic COVID-19 patients and “SARS-CoV-2 aborters,” are ideal targets for inclusion in a next-generation CoV vaccine^33–36^. It is likely that the rapid induction of local mucosal antigen-specific CD4^+^ and CD8^+^ T cells by early expressed NSP-2, NSP-14, or Nucleoprotein contributed to the fast control of virus replication and reduced lung pathology in vaccinated hamsters. Furthermore, the Nucleoprotein is the most abundant viral protein and one of the most predominantly targeted antigens by T cells in individuals with less severe COVID-19 disease^39,40^. Our results also align with a previous report demonstrating that Nucleoprotein-specific T cell responses were associated with control of SARS-CoV-2 in the upper airways and with improved lung pathology before seroconversion^62^. The hamsters receiving the combination mRNA/LNP vaccine showed improved lung pathology compared to mock-vaccinated animals, which exhibited severe injury characterized by reduced lung vacuoles, epithelial denudation, vascular leak, hyaline membrane formation, neutrophilic infiltration, septal thickening, and intraluminal proteinaceous material. Histopathologic interpretation was grounded in established DAD criteria^63–66^, with blinded veterinary pathologist review and quantitative assessment across multiple lung regions to distinguish true injury from fixation artifact. These findings are consistent with prior SARS-CoV-2 animal model studies reporting alveolar thickening, hyaline membranes, inflammatory infiltrates, and edema^46,47,67–73^. Notably, pneumocyte cytoplasmic vacuolation, while described in human DAD autopsy material, is nonspecific and should be interpreted within the broader injury pattern rather than as a unique viral signature^74–77^. Together, these data support a standardized histologic approach for evaluating SARS-CoV-2-associated pulmonary pathology.

Following virus challenge, mock-vaccinated animals inoculated with Delta exhibited markedly higher viral titers than those inoculated with the ancestral Washington variant (WA1) or Omicron XBB.1.5. This likely reflects Delta’s superior replication fitness and enhanced cell entry capacity, driven in part by Spike mutations such as P681R, which augment furin cleavage efficiency, TMPRSS2-dependent membrane fusion, and syncytia formation^78^, properties that collectively underlie its heightened pathogenicity relative to other variants. WA1, as an early ancestral strain, lacks these adaptations and therefore serves as a lower-replication baseline in this comparison. XBB.1.5, by contrast, represents a divergent evolutionary trajectory: rather than acquiring fusogenic or replication-enhancing mutations analogous to those of Delta, this subvariant evolved primarily toward optimized ACE2 receptor binding and immune evasion^79,80^. As a result, despite its extensive immune escape capacity, XBB.1.5 appears less efficient at driving high-titer replication in the respiratory tract under the conditions tested here. Together, these findings highlight that peak viral load in the upper respiratory tract is shaped not only by the host’s immune status but also by the infecting variant’s intrinsic replication biology, a distinction with important implications for interpreting cross-variant vaccine efficacy data.

The 10 selected protein antigens in this study are highly conserved across all VOCs, including the highly transmissible and immune-evasive Omicron subvariants KP.3, NB.1.8.1, and LP.8.1, which are currently spreading worldwide. In contrast, the Spike protein is highly mutated in these variants, with a total of 346 mutations since the ancestral Wuhan strain. This includes up to 60 new mutations in BA.2.86, JN.1, KP.3, NB.1.8.1, and LP.8.1, which are currently spreading worldwide as subvariants of the original COVID-19 virus. The sequences of the protective T cell antigens NSP-2, NSP-14, and Nucleoprotein remain highly conserved in BA.2.86 and JN.1. Of note, the sequence of NSP-14 is fully conserved (100%) in all variants and sub-variants, including the BA.2.86, JN.1, KP.3, NB.1.8.1, and LP.8.1 supporting the vital function of NSP-14 protein in the SARS-CoV-2 life cycle^81–86^. NSP-4 (527 aa) is a bifunctional protein; the N-terminal domain has a methyltransferase function required for virus replication^81–83^, and the C-terminal domain has a proofreading exonuclease function in viral RNA 5′ capping and facilitates viral mRNA stability and translation^82,84–86^. NSP-2 (638 aa) is a multi-subunit RNA-dependent RNA polymerase (RdRp) that is involved in replication and RNA synthesis^87,88^. Nucleoprotein (419 aa) plays a vital role in identifying and facilitating viral RNA packaging and in regulating viral replication and transcription^89^. The critical role these viral proteins play in CoV replication, and their conservation in SARS-CoV-1, MERS-CoV, SARS-CoV-2, and animal SL-CoVs from bats, pangolins, civet cats, and camels, make them ideal targets for a next-generation vaccine capable of ending the current COVID-19 pandemic and preventing future CoV outbreaks.

Although NSP2- and NSP14-vaccinated animals showed similar levels of SARS-CoV-2 viral RNA, they had similar protection against disease severity^90^. Since RT-qPCR was used to detect viral genome copies that can come from infectious virus, noninfectious particles, infected-cell debris, or residual RNA, the viral RNA burden does not always correlate directly with productive infection, tissue damage, or clinical outcomes, such as weight loss^90^. Because NSP2 and NSP14 are internal viral proteins with varying expression kinetics, NSP2- and NSP14-based vaccines likely prevent infection by inducing antiviral T-cells against infected cells, with varying kinetics. As a result, NSP2- and NSP14-based vaccines may differ in how induced T-cells reduce lung inflammation and prevent weight loss^90^. Besides, their immunogenicity and protective efficacy, NSP-2 and NSP-14 are functionally distinct antigens; NSP-2 is involved in host-cell interactions, whereas NSP-14 has key enzymatic roles in viral RNA proofreading and RNA capping, including 3′–5′ exonuclease and N7-methyltransferase activities^91^. In contrast, NSP-14 has been linked to modulation of host antiviral and inflammatory pathways, including interferon-related effects, which could affect disease severity^92^.

Pairwise global alignment of amino acid sequences revealed 100% identity for NSP-2 (638 residues) across the SARS-CoV-2 Washington, Delta, and Omicron variants. For NSP-14 (527 residues), pairwise comparisons showed 99.81% sequence identity between Washington and Delta (A394V substitution) and between Washington and Omicron (I42V substitution), each differing by a single amino acid. The Delta/Omicron NSP-14 comparison yielded 99.62% identity, with two amino acid differences (I42V and V394A). For the nucleoprotein, the Washington and Delta sequences each encode 419 residues, whereas the Omicron sequence encodes 416 residues. Washington and Delta nucleoprotein sequences share 99.04% identity, differing at four positions (D63G, R203M, G215C, D377Y). Washington and Omicron nucleoprotein sequences share 98.32% identity, with seven differences: three deletions (P13L, E31del, R32del, S33del) and four substitutions (R203K, G204R, S413R). The Delta/Omicron nucleoprotein comparison showed the greatest divergence among the three pairwise comparisons at 97.61% identity, with differences at 10 positions (P13L, E31del, R32del, S33del, G63D, M203K, G204R, C215G, Y377D, S413R). By contrast, the Spike glycoprotein exhibited substantially greater inter-variant divergence: Washington and Delta Spike proteins differ by 0.63%, Washington and Omicron by 2.36%, and Delta and Omicron by 2.36%. Collectively, these data demonstrate that NSP-2, NSP-14, and the nucleoprotein are highly conserved across antigenically distinct SARS-CoV-2 variants, supporting the rationale that a T-cell antigen-based vaccine targeting these proteins can confer broad cross-protective efficacy against the ancestral Washington strain, the highly pathogenic Delta variant, and the highly transmissible Omicron variant.

Previous preclinical and clinical studies have supported the hypothesis of targeting T cell antigens to broaden vaccine-induced immunity. These include vaccines based on Nucleoprotein, using various delivery technologies, such as recombinant protein^93^, DNA vaccines^94^, mRNA^51,95^, and viral vectors^96–98^. In these cases, Nucleoprotein-specific T cell responses and protective immunity have been demonstrated in animal models, and immunogenicity has been demonstrated in humans. Another approach has been to target specific human T-cell epitopes using in silico methods to identify them (Vahed, in Press). One vaccine candidate is based on numerous CD8^+^ T cell epitopes linked in tandem, delivered by a self-replicating mRNA vaccine^3,99–101^. Another candidate targets discrete epitopes and segments of T cell antigens, delivered by a conventional base-modified mRNA^102^. Antigen-specific immune responses and protective immunity have been demonstrated in animal models, and both vaccine candidates have been evaluated in humans in clinical trials. One potential limitation of epitope-based strategies is the breadth of immunity, in terms of both the number of epitopes presented to the immune system and the degree of coverage across the diversity of human HLA haplotypes. Epitope-specific vaccines are also susceptible to immune evasion, as mutations may create mismatches between vaccine epitopes and circulating viral strains, thereby rendering the vaccine ineffective. In contrast, the inclusion of multiple whole T-cell antigens provides substantial redundancy of epitopes and enables determinant selection based on individual HLA types. Using computational and informatics approaches, we identified hundreds of putative human CD4 and CD8 epitopes across the three T cell antigens, collectively covering more than 99% of human HLA diversity (*not shown*).

As with most diseases, no single animal model can fully replicate immune responses to SARS-CoV-2 infection and COVID-19 as they occur in humans; however, studies conducted in the hamster model, which develops infection and COVID-19-like symptoms similar to those observed in patients, may yield a vaccine that would confer protection in humans. Moreover, the strong positive linear correlation between the high magnitude of IFN-γ-producing CD4^+^ and CD8^+^ T cells specific for NSP-2, NSP-14, and Nucleoprotein common antigens and the “natural protection” observed in unvaccinated asymptomatic COVID-19 patients likely supports the protective efficacy of vaccines that incorporate these three common T cell antigens. Moreover, we have shown T-cell-dependent protection against in the “humanized” HLA double-transgenic mouse model, which expresses human HLA-A*0201 and HLA-DR*0101 in place of the corresponding mouse MHC class I and class II molecules^103,104^. The HLA-A*0201 allele was chosen because it is highly represented (>50%) in the human population, regardless of race or ethnicity^105–107^. This novel “humanized” hACE-2/HLA-A*0201/HLA-DR-1 triple transgenic mouse model has three advantages: (1) it is susceptible to human SARS-CoV-2 infection. SARS-CoV-2 replication in the lungs was observed in hACE2 transgenic mice following intranasal inoculation. Infection begins in airway epithelia, with subsequent alveolar involvement and extrapulmonary virus spread to the brain; (2) it then develops human COVID-19-like symptoms (i.e., pneumonia, lung histopathology, weight, smell, and taste loss)^108^. Upregulation of proinflammatory cytokines and chemokines^109–112^ (i.e., cytokine storm) in both the lungs and the brain leads to death^113–116^; and (3) it mounts human-like HLA-restricted CD4^+^ and CD8^+^ T cell responses directed toward human T cell epitopes.

Several limitations of the present studies warrant consideration. Although we demonstrated cross-protective efficacy of the multi-antigen CoV vaccine against multiple VOCs, important knowledge gaps remain. First, the protective efficacy of the combined multi-antigen CoV vaccine was evaluated in immunologically naïve hamsters. To more closely model real-world immune landscapes, future studies will assess the efficacy of the mRNA/LNP combination vaccine in hamsters with pre-existing Spike-specific or SARS-CoV-2-specific immunity^117^. Second, given that NSP-2, NSP-14, and the nucleoprotein harbor sequences of high homology between SARS-CoV-2 and common cold coronaviruses (CCCs), the contribution of cross-reactive T cells elicited by the combined multi-antigen CoV vaccine should be investigated in animals previously infected with one of the four major CCC strains (α-CCC-229E, α-CCC-NL63, β-CCC-HKU1, or β-CCC-OC43). Third, to establish broad-spectrum efficacy, the NSP-2, NSP-14, and nucleoprotein T-cell antigen-based vaccine will be evaluated against MERS-CoV and SARS-CoV in subsequent studies. Fourth, as the combined mRNA/LNP vaccine substantially reduced viral load in the upper respiratory tract, future work will also examine whether this protection extends to a reduction in viral transmission^7^. If so, this would be a highly desirable attribute of a next-generation vaccine capable of blocking the transmission cycle. Finally, this report shows that the combination vaccine elicited lung-resident antigen-specific T_FH_, T_CYT,_ and T_EFF_ cells that may have contributed to eliminating lung-infected epithelial cells and interfered locally with virus replication in the lungs, consistent with reports showing cross-reactive memory CD4^+^ and CD8^+^ T cells alone (without antibodies) may have protected SARS-CoV-2-infected patients with B cell depletion from severe disease^118–120^, and in non-human primate studies showing that SARS-CoV-2-specific T cells reduced viral loads^121^. However, these may not be the only underlying immune mechanisms responsible for the observed cross-protection. Because immunological reagents and monoclonal antibodies (mAbs) are limited in the hamster model, a better understanding of the B and T cell mechanisms of protection induced by the combined mRNA/LNP vaccine would be enhanced by studying it in mice. Our novel ACE2/HLA triple-transgenic mouse model, which expresses the human HLA-DR and A*0201 alleles, is well-suited for examining the role of T effector cells, dissecting early protein expression, antigen presentation, and the stimulation of innate and inflammatory responses.

In summary, this pre-clinical study in the hamster model presents pathological, virological, and immunological evidence that a Spike-, NSP-2-, NSP-14-, and Nucleoprotein-based combination mRNA/LNP vaccine induced durable, stronger, and broader protection against infection and disease caused by various VOCs compared to the Spike mRNA/LNP vaccine alone, thereby creating a superior vaccine than the current standard of care. The observed protection induced by the combined vaccine was associated with the induction of both Spike-specific neutralizing antibodies and T cell antigen-specific lung-resident CD4 and CD8 T_FH_, T_CYT_, and T_EFF_ cells, which persist for more than 1-year post-vaccination. These attributes hold promise for a superior next-generation broad-spectrum CoV vaccine and warrant evaluation in human clinical trials.

## Methods

### Human study population cohort and HLA genotyping

Between January 2020 and December 2023, more than 1100 unvaccinated patients with mild to severe COVID-19 were enrolled at the University of California, Irvine Medical Center under an approved Institutional Review Board (IRB) protocol (IRB#2020-5779). Written informed consent was obtained from all patients before their inclusion. A positive RT-PCR test defined SARS-CoV-2 positivity on a respiratory tract sample. The unvaccinated COVID-19 patients were enrolled throughout the pandemic irrespective of SARS-CoV-2 variants of concern they are exposed to: The ancestral Washington variant (USA-WA1/2020), alpha, beta, gamma, the highly pathogenic Delta variant (B.1.617.2), or the omicron subvariants B.1.1.529, BA.2.86, XBB1.5, EG.5, HV.1, JN.1, KP.3, NB.1.8.1, and LP.8.1. Patients were genotyped by PCR for class I HLA-A*02:01 and class II HLA-DRB1*01:01: and ended up with 147 that were HLA-A*02:01^+^ or/and HLA-DRB1*01:01^+^. The 147 patients were from mixed ethnicities (Hispanic (28%), Hispanic Latino (22%), Asian (16%), Caucasian (13%), mixed Afro-American and Hispanic (8%), Afro-American (5%), mixed Afro-American and Caucasian (2%), Native Hawaiian and Other Pacific Islander descent (1%). Six percent of the patients did not disclose their race or ethnicity. The disease severity of the COVID-19 patients included in this study was defined based on earlier studies^41,122^.

### Peptide synthesis

Peptide-epitopes from 12 SARS-CoV-2 proteins, including 16 9-mer long CD8^+^ T cell epitopes and 13 15-mer long CD4^+^ T cell epitopes that were selected as described previously^5^. Peptides were synthesized (21^st^ Century Biochemicals, Inc., Marlborough, MA), and the purity of peptides was determined by both reversed-phase high-performance liquid chromatography and mass spectroscopy to be over 95%.

### Human peripheral blood mononuclear cells and T cell assays

PBMCs from COVID-19 patients were isolated from the blood using Ficoll (GE Healthcare) density gradient media and transferred into 96-well plates at a concentration of 2.5 × 10^6^ viable cells per ml in 200 µl (0.5 × 10^6^ cells per well) of RPMI-1640 media (Hyclone) supplemented with 10% (v/v) FBS (HyClone), Sodium Pyruvate (Lonza), L-Glutamine, Nonessential Amino Acids, and antibiotics (Corning). A fraction of the blood was kept separate to perform HLA genotyping on only individuals who were positive for HLA-A*02:01 and DRB1*01:01. Fresh blood samples were collected from COVID-19 patients during the acute phase of infection (days 3–8) from subjects admitted to the UC Irvine hospitals. For non-admitted patients, fresh blood was collected on day 2 after the onset of COVID-19-like symptoms and when they visited the hospital for a check-up. Fresh PBMCs were used in this study, as they generally have higher viability and functionality compared to frozen PBMCs. Freezing and thawing can cause cell damage and loss of T-cell function, potentially affecting the accuracy and reliability of experimental results. The average timeline for fresh PBMC collection was 4.8 days after the onset of COVID-19 symptoms (Supplementary Fig. 1). Fresh PBMCs were used in this study, as they generally have higher viability and functionality compared to frozen PBMCs. Freezing and thawing can cause cell damage and loss of T-cell function, potentially affecting the accuracy and reliability of experimental results. Subsequently, cells were stimulated with 10 µg/ml of each one of the 29 individual T cell peptide-epitopes (16 CD8^+^ T cell peptides and 13 CD4^+^ T cell peptides) and incubated in a humidified chamber with 5% CO_2_ at 37 °C. Post-incubation, cells were stained for flow cytometry or transferred to IFN-γ ELISpot plates (Supplementary Fig. 1A). The same isolation protocol was followed for HD samples obtained before the COVID-19 pandemic (2018). Ficoll was kept frozen in liquid nitrogen in FBS and DMSO (10%). After thawing, HD PBMCs were stimulated similarly for the IFN-γ ELISpot. Sequence details of all the 9-mer CD4^+^ T cell epitopes and 15-mer CD8^+^ T cell epitopes are provided in Supplementary Figs. 2 and 3.

### Human ELISpot assay and flow cytometry

We assessed CD4^+^ and CD8^+^ T-cell response against conserved SARS-CoV-2-derived class-II restricted epitopes by IFN-γ ELISpot in COVID-19 patients representing different disease severity categories (Supplementary Fig. 1A). All ELISpot assays were performed as described earlier^41^. Similarly, surface marker detection and flow cytometry analysis were performed on patients 72 h after stimulation with each SARS-CoV-2 class I- or class II-restricted peptide^41^. The gating strategy for flow cytometry is detailed in Supplementary Fig. 1B.

### Enzyme-linked Immunosorbent assay

Spike-specific IgG antibody in the serum of hamsters immunized with Spike 2 proline (1 µg) compared to Spike 6 proline (1 µg) was detected by ELISA. Blood was collected from immunized hamsters on Day 40 post-immunization, prior to challenge with SARS-CoV-2. The 96-well plates (Dynex Technologies, Chantilly, VA) were coated with 100 ng of Spike (S1 + S2) (SinoBiological) per well and kept at 4 °C overnight. The plates were subsequently washed thrice with PBS and blocked with 3% BSA (in 0.1% PBST) for 2 h at 37 °C. After blocking, the plates were incubated with serially diluted sera (100 μl/well, twofold dilutions) for 2 h at 37 °C. Bound serum antibodies were detected using HRP-conjugated goat anti-mouse IgG and the chromogenic substrate TMB (Thermo Fisher, Waltham, MA). The cutoff for seropositivity was set at the mean value plus 3 standard deviations (3SD) of HBc-S control sera.

### Viruses

SARS-CoV-2 specific to six variants, namely (*i*) SARS-CoV-2-USA/WA/2020 (Batch Number: G2027B); (*ii*) Delta (B.1.617.2) (isolate h-CoV-19/USA/MA29189; Batch number: G87167), (*iii*) Omicron (XBB1.5) (isolate h-CoV-19/USA/FL17829; Batch number: G76172), were procured from Microbiologics (St. Cloud, MN). Omicron KP.3 (Isolate hCoV-19/USA/NJ-GBW-GKISBBBB88291/2024) was procured from the BEI resources. The initial viral stocks were propagated to generate high-titer virus. Vero E6 (ATCC-CRL1586) cells were used for this purpose. Procedures were completed using aseptic technique under BSL-3 containment.

### mRNA synthesis

Sequences of Spike and 10 T cell non-Spike antigens were derived from the SARS-CoV-2 Omicron sub-variant BA.2 (NCBI GenBank accession number OM617939) Nucleoside-modified mRNAs expressing SARS-CoV-2 full-length of prefusion-stabilized Spike protein with two or 6 proline mutations (mRNA-S-2P and mRNA-S-6P (nucleotide range: 23,008–25,117 bp) and part or full-length 10 highly conserved non-Spike T cell antigens (NSP-2 (nucleotide range: 469–1867 bp), NSP-3 (nucleotide range: 3906–8289 bp), NSP-4 (nucleotide range: 8290–9789 bp), NSP-5-10 (nucleotide range: 9790–13,167 bp), NSP-12/RdRP (nucleotide range: 13,168–13,177 bp), NSP-14 (nucleotide range: 13,168–13,177 bp), ORF7a/b (nucleotide range: 27,688–27819 bp), Membrane (nucleotide range: 26,455–27,123 bp), Envelope (nucleotide range: 26,177–26,402 bp), and Nucleoprotein (nucleotide range: 28,206–29,454 bp))) were synthesized by in vitro transcription using T7 RNA polymerase (MegaScript, Thermo Fisher Scientific, Waltham, MA) on linearized plasmid templates, as reported^42,123^. Modified mRNA transcript with complete substitution of Pseudo-U was synthesized by TriLink Biotechnologies using proprietary CleanCap® technology. The synthesized polyadenylated (80A) mRNAs were subjected to DNase and phosphatase treatment, followed by Silica membrane purification. Finally, the synthesized mRNA was packaged as a 1.00 ± 6% mg/mL solution in 1 mM Sodium Citrate, pH 6.4. Purified mRNAs were analyzed by agarose gel electrophoresis and were kept frozen at −20 °C. Formulated mRNAs were prepared at varying RNA concentrations (1 μg/μL) and stored at −80 °C.

### LNP formulation

The mRNAs were formulated into LNPs using an ethanolic lipid mixture of ionizable cationic lipid and an aqueous buffer system^124^. A customized GenVoy-ILM-based lipid nanoparticle containing an ionizable cationic lipid and with compositions of phospholipid, cholesterol, and stabilizer was provided by Precision NanoSystems (PNI) (currently Cytiva) (Vancouver, BC, Canada). The LNP (Identifier IL00V41) comprised a unique ionizable phospholipid, DOPE (1,2-dioleoyl-sn-glycero-3-phosphoethanolamine), and a “higher” amount of ionizable lipid; its structure is proprietary to Cytiva^124^. The physical properties associated with this LNP are Particle Size (94 nm), PDI (0.154), Encapsulation Efficiency (98.4%), and Zeta Potential (-3). Formulated mRNA-LNPs were stored at −80 °C for animal immunizations^124^.

### Confirmation of protein expression by mRNA

The expression of the target viral protein by the vaccines was confirmed in HEK293T [American Type Culture Collection (ATCC), CRL-3216] cells before testing in animal experiments, and 10^6^ cells were plated in 500 µl of culture medium in a 6-well plate on Day 0. Once the cells reached confluency, *HEK*293T cells in six-well plates were either directly transfected with 2 μg of mRNA-LNP or transfected with LNP alone. A transfection mix for mRNA was prepared, and cells were transfected according to the Lipofectamine MessengerMAX Transfection Reagent-specific protocol (Thermo Fisher Scientific, Catalog # LMRNA001).

### Comparative analysis of overlapping T-cell epitopes in the vaccine and SARS-CoV-2 variants

MHC-I binding prediction and MHC-II Binding prediction tools hosted by the IEDB were used for a comparative analysis of overlapping T-cell epitopes in the vaccine candidates used in this study and those of SARS-CoV-2 variants (SARS-CoV-2 WA/2020, Delta B.1.617.2, and Omicron XBB.1.5) (Supplementary Data 1).

### Hamster immunization and SARS-CoV-2 variants challenge

The mRNA/LNP vaccines were evaluated in the outbred golden Syrian hamster model for protection against three SARS-CoV-2 variants and subvariants (Washington, Delta, and Omicron). The Institutional Animal Care and Use Committee approved animal model usage experiments at the University of California, Irvine (Protocol number AUP-22-086). The recommendations in the *Guide for the Care and Use of Laboratory Animals*, published by the National Institutes of Health, are used for performing animal experiments. The sample size for each animal study (*n* = 6 per group) was determined by power analysis, demonstrating that five hamsters per group were sufficient to achieve a power greater than 80%.

For variants and subvariants (Washington, Delta, and Omicron challenge), four groups of 6- to 8-week-old male golden Syrian hamsters (6 per group), strain HsdHan: AURA (Envigo, 8901 M), were vaccinated with a double blind approach intramuscularly with individual or combined mRNA/LNP (1 μg, 5 μg, or 10 μg per dose as indicated in figures) on day 0 (prime) and day 21 (boost). Hamsters that received PBS alone were used as mock-immunized controls (*Saline*, *Mock*, *n* = 6). The mRNA/LNP vaccines and saline control were administered in 100 μl per injection. Serum samples were collected from all hamsters before the viral challenge to measure vaccine-induced neutralizing antibodies. Three weeks after booster vaccination (week 6), the hamsters were transferred to the ABSL-3 facility and intranasally challenged with the SARS-CoV-2 Delta variant (1 × 10^5^ plaque-forming units [pfu]) or with the Washington or Omicron strain (2 × 10^5^ pfu). At the indicated time points, throat swab samples and corresponding lung tissue samples were collected for analyses of vaccine-induced protection. Hamsters were monitored daily to evaluate vaccine-induced protection from body weight loss. Hamsters were anesthetized with Isoflurane (Fluriso^TM^, VETone) and euthanized with CO_2_, as approved by the IACUC (Protocol # AUP-22-086).

### Hamster lung mononuclear cells and T cell assays

Single-cell suspensions were generated from both lungs of each hamster by collagenase treatment (8 mg/ml) for 1 h. The resulting cell suspensions were then passed through a 70-μm cell strainer to obtain a uniform single-cell suspension, which was subsequently used to stimulate peptide pools. Cells were stimulated for 48 h at 37 °C in a CO_2_ incubator. Subsequently, the stimulated cells were stained for CD4 (APC, clone GK1.5-BioLegend), CD8 (FITC, anti-rat clone 341-eBioScience), CXCR5 (PerCP, clone L138D7-BioLegend), and CD69 (PECy7, anti-human clone FN50-BioLegend). Surface staining was performed by adding mAbs against various cell markers to a total of 1 × 10^6^ cells in PBS containing 1% FBS and 0.1% sodium azide, followed by incubation for 45 min at 4 °C. The cells were washed three times with FACS buffer (PBS, 1% FBS, and 0.1% sodium azide) and fixed in PBS containing 2% paraformaldehyde (Sigma-Aldrich, St Louis, Missouri, USA). A total of 100,000 lymphocyte-gated PBMCs were acquired by Fortessa X20 (Becton Dickinson, Mountain View, California, USA) and analyzed using FlowJo software (v10.10.0). For the intracellular staining, after surface staining, the lymphocytes were treated with Cytofix/Cytoperm (Becton Dickinson, USA), and stained for 45 min at 4 °C with IFN-γ (PE, clone XMG1.2-BioLegend), TNF-α (APC-Cy7, clone MP6- XT22 - BD), and GzmB (BV421, clone QA16A02-BioLegend). The cells were washed three times with FACS buffer and fixed in PBS containing 2% paraformaldehyde (Sigma-Aldrich, St Louis, Missouri, USA). A total of 100,000 lymphocyte-gated PBMCs were acquired on the Fortessa X20 (Becton Dickinson, Mountain View, California, USA) and analyzed using FlowJo software (v10.10.0).

### Neutralizing assay

Serum neutralizing activity was examined, as previously reported^59,125^. Briefly, the assays were performed using Vero E6 cells (ATCC, CRL-1586). Briefly, serum samples were heat-inactivated and serially diluted threefold (initial dilution, 1:10), followed by incubation with 100 pfu of either wild-type SARS-CoV-2 (USA-WA1/2020) or the Delta strain for 1 h at 37 °C. The serum-virus mixtures were applied to Vero E6 cell monolayers in 96-well plates and incubated at 37 °C for 1 h. The plates were washed with DMEM, and the monolayer cells were overlaid with 200 μL of minimum essential medium (MEM) containing 1% (w/v) methylcellulose, 2% fetal bovine serum (FBS), and 1% penicillin-streptomycin. Cells were then incubated for 24 h at 37 °C. Vero E6 monolayers were washed with PBS and fixed with 250 μl of pre-chilled 4% formaldehyde for 30 min at room temperature, followed by aspiration removal of the formaldehyde solution and twice with PBS. The cells were permeabilized using 0.3% (wt/vol) hydrogen peroxide in water. The cells were blocked with 5% nonfat dried milk, followed by the addition of 100 μL of diluted anti-SARS-CoV-2 antibody (1:1000) to all wells of the microplates for 1–2 h at room temperature. This was followed by incubation with a diluted anti-rabbit IgG conjugate (1:2000) for 1 h at room temperature. The plate was washed and developed with TrueBlue substrate, and foci were counted using an ImmunoSpot analyzer. Each serum sample was tested in duplicate.

### Depletion of CD4^+^ and CD8^+^ T-cell subsets

Rat IgG2b monoclonal antibodies (mAbs) specific for mouse CD4 (Clone Gk1.5) and mouse CD8 (Clone 2.43) were used for in vivo T cell depletion. hACE2 transgenic mice were injected intraperitoneally (i.p.) with 300 µg of anti-mouse CD4 and/or anti-mouse CD8α mAbs. Mice were subjected to this injection 4 and 2 days before the challenge with the SARS-CoV-2 B.617.2 (Delta) variant. Mice were subsequently followed till Day 14 post-infection to monitor Physical estimation (Weight loss and Viral Titration), comparison of mice immunized with a combination of Nucleoprotein + NSP2 + NSP14 (at 1 μg each) and subjected to CD8, CD4, and CD4 + CD8 T-cell depletion vs immunized mice in which the T cells were not depleted. Immunized mice infected with SARS-CoV-2 Delta variant (at 5 × 10^5^ pfu).

### RNA extraction and RT-PCR quantification of viral RNA copies

The oropharyngeal swab samples from hamsters and mice were analyzed for SARS-CoV-2-specific RNA by quantitative RT-PCR (qRT-PCR). As recommended by the Centers for Disease Control and Prevention (CDC), we used ORF1ab-specific primers (forward: 5′-CCCTGTGGGTTTTACACTTAA-3′ and reverse: 5′-ACGATTGTGCATCAGCTGA-3′) to detect the viral RNA level. PCR reactions (10 µl) contained primers (10 µM), cDNA sample (1.5 µl), SYBR Green reaction mix (5 µl), and molecular-grade water (2.5 µl). PCR cycling conditions were as follows: 95 °C for 3 min, 45 cycles of 95 °C for 5 s, and 60 °C for 30 s. For each RT-PCR run, a standard curve was generated using an RNA standard (Armored RNA Quant^®^) to quantify the absolute number of viral RNA copies in the throat swabs.

### Lung histopathology

Lungs were inflated in situ with 10% neutral-buffered formalin at physiologic pressure before fixation to minimize artifactual alveolar collapse^126^, then transferred to 70% ethanol and processed using standardized histopathologic procedures to preserve tissue architecture. Fixed tissues were paraffin-embedded, sectioned at 8 μm, deparaffinized, rehydrated, and stained with H&E. All slides were evaluated in a blinded manner by a board-certified veterinary pathologist. Histopathologic assessment was performed using established criteria for DAD, including evaluation of alveolar septal thickening, inflammatory cell infiltration, proteinaceous exudate, hyaline membrane formation, and pneumocyte injury^126–128^, consistent with American Thoracic Society guidelines for experimental acute lung injury scoring. Septal thickening was identified by widening and increased cellularity of alveolar walls with narrowing of airspaces. Inflammatory infiltration was assessed by increased hematoxylin-positive inflammatory cells within septa, perivascular regions, or airspaces. Proteinaceous exudate was identified as amorphous eosinophilic material within alveolar or airway lumens. Hyaline membranes were identified as linear eosinophilic membrane-like deposits lining alveolar surfaces. Pneumocyte injury was assessed by epithelial attenuation, denudation, sloughing, or reactive pneumocyte morphology. Cytoplasmic vacuolation in pneumocytes was noted but interpreted within the broader context of DAD, recognizing its nonspecific nature despite prior reports in SARS-CoV-2-infected human and animal tissues^66,72,75^ (Figs. 4, 5 and Supplementary Fig. 6), in accordance with previously described SARS-CoV-2 animal models^68,69^.

### Data and code availability

Human-specific SARS-CoV-2 complete genome sequences were retrieved from the GISAID database, whereas the SARS-CoV-2 sequences for bats, pangolin, civet cats, and camels were retrieved from the NCBI GenBank. The genome sequence accession numbers retrieved from NCBI GenBank are listed in detail in our earlier publication^5,122^. Additional raw data are available from the corresponding author upon reasonable request. All data supporting the findings of this study are available within the paper and its Supplementary Information.

### Statistics

Statistical analysis was performed using GraphPad Prism 10.0 software (GraphPad Software, La Jolla, CA). Nonparametric tests were used throughout this paper for statistical analysis. Data were expressed as the mean ± SD. Comparisons among groups were performed using the Mann–Whitney test (two-group comparisons). Two-tailed *P* values were denoted, and *P* values < 0.05 were considered significant.

## Supplementary information

Supplementary Figures

ARRIVE Compliance Questionnaire

Supplementary Data 1

## References

[1] Prakash, S. et al. Cross-protection induced by highly conserved human B, CD4 (+,) and CD8 (+) T cell epitopes-based coronavirus vaccine against severe infection, disease, and death caused by multiple SARS-CoV-2 variants of concern. *Front. Immunol.* **29**, 45–56 (2024). 10.3389/fimmu.2024.1328905 PMC10839970 38318166

[2] Dhanushkodi, N. R. et al. Anti-viral and anti-inflammatory therapeutic effect of RAGE-Ig protein against multiple SARS-CoV-2 variants of concern demonstrated in K18-hACE2 mouse and Syrian golden hamster models. *J. Immunol.* **212**, 1–10 (2024). 10.4049/jimmunol.2300392 38180084

[3] Zayou, L. et al. A multi-epitope/CXCL11 prime/pull coronavirus mucosal vaccine boosts the frequency and the function of lung-resident memory CD4(+) and CD8(+) T cells and enhanced protection against COVID-19-like symptoms and death caused by SARS-CoV-2 infection. *J. Virol.* **97**, 1–13 (2023). 10.1128/jvi.01096-23 PMC10734477 38038432

[4] Evans, J. P. & Liu, S. L. Challenges and prospects in developing future SARS-CoV-2 vaccines: overcoming original antigenic sin and inducing broadly neutralizing antibodies. *J. Immunol.* **211**, 1459–1467 (2023). 10.4049/jimmunol.2300315 37931210

[5] Prakash, S. et al. Genome-wide B cell, CD4(+), and CD8(+) T cell epitopes that are highly conserved between human and animal coronaviruses, identified from SARS-CoV-2 as targets for preemptive pan-coronavirus vaccines. *J. Immunol.* **206**, 2566–2582 (2021). 10.4049/jimmunol.2001438 PMC8722450 33911008

[6] Pedersen, S. F. & Ho, Y. C. SARS-CoV-2: a storm is raging. *J. Clin. Investig.* **130**, 2202–2205 (2020). 10.1172/JCI137647 PMC7190904 32217834

[7] Sharma, S. et al. Updated vaccine protects against SARS-CoV-2 variants including Omicron (B.1.1.529) and prevents transmission in hamsters. *Nat. Commun.* **13**, 6644 (2022). 10.1038/s41467-022-34439-7 PMC9636174 36333374

[8] Sunagar, R., Singh, A. & Kumar, S. SARS-CoV-2: immunity, challenges with current vaccines, and a novel perspective on mucosal vaccines. *Vaccines* **11**, 849 (2023). 10.3390/vaccines11040849 PMC10143972 37112761

[9] Washington, N. L. et al. Emergence and rapid transmission of SARS-CoV-2 B.1.1.7 in the United States. *Cell* **184**, 2587–2594 e2587 (2021). 10.1016/j.cell.2021.03.052 PMC8009040 33861950

[10] Konings, F. et al. SARS-CoV-2 Variants of Interest and Concern naming scheme conducive for global discourse. *Nat. Microbiol.* **6**, 821–823 (2021). 10.1038/s41564-021-00932-w 34108654

[11] Bellocchi, M. C. et al. Frequency of atypical mutations in the spike glycoprotein in SARS-CoV-2 circulating from July 2020 to July 2022 in Central Italy: a refined analysis by next generation sequencing. *Viruses* **15**, 1711 (2023). 10.3390/v15081711 PMC10458583 37632054

[12] Beale, S. et al. Anti-nucleocapsid and anti-spike antibody trajectories in people with post-covid condition versus acute-only infections: a nested longitudinal case-control study within the Virus Watch prospective cohort. *Nat. Commun.* **16**, 3686 (2025). 10.1038/s41467-025-58766-7 PMC12006544 40246813

[13] Davis, H. E., McCorkell, L., Vogel, J. M. & Topol, E. J. Long COVID: major findings, mechanisms and recommendations. *Nat. Rev. Microbiol.* **21**, 133–146 (2023). 10.1038/s41579-022-00846-2 PMC9839201 36639608

[14] Al-Aly, Z. et al. Long COVID science, research and policy. *Nat. Med.* **30**, 2148–2164 (2024). 10.1038/s41591-024-03173-6 39122965

[15] Hou, Y. et al. Global Prevalence of Long COVID, Its Subtypes, and Risk Factors: An Updated Systematic Review and Meta-analysis. *Open Forum Infect Dis.* **12**, ofaf533 (2025). 10.1093/ofid/ofaf533 PMC12461872 41018705

[16] Cevik, M., Grubaugh, N. D., Iwasaki, A. & Openshaw, P. COVID-19 vaccines: keeping pace with SARS-CoV-2 variants. *Cell* **184**, 5077–5081 (2021). 10.1016/j.cell.2021.09.010 PMC8445744 34534444

[17] Yisimayi, A. et al. Repeated Omicron exposures override ancestral SARS-CoV-2 immune imprinting. *Nature* **625**, 148–156 (2024). 10.1038/s41586-023-06753-7 PMC10764275 37993710

[18] Renner, T. M. et al. Tuning the immune response: sulfated archaeal glycolipid archaeosomes as an effective vaccine adjuvant for induction of humoral and cell-mediated immunity towards the SARS-CoV-2 Omicron variant of concern. *Front. Immunol.* **14**, 1182556 (2023). 10.3389/fimmu.2023.1182556 PMC10140330 37122746

[19] Ma, Q. et al. SARS-CoV-2 bivalent mRNA vaccine with broad protection against variants of concern. *Front. Immunol.* **14**, 1195299 (2023). 10.3389/fimmu.2023.1195299 PMC10244545 37292197

[20] Rubin, E. J., Baden, L. R., Marks, P. & Morrissey, S. Audio interview: the FDA and Covid-19 vaccines. *N. Engl. J. Med.* **387**, e60 (2022). 10.1056/NEJMe2214911 36383718

[21] Chowell, G. & Mizumoto, K. The COVID-19 pandemic in the USA: What might we expect?. *Lancet* **395**, 1093–1094 (2020). 10.1016/S0140-6736(20)30743-1 PMC7270048 32247381

[22] The, L. The COVID-19 pandemic in 2023: far from over. *Lancet* **401**, 79 (2023). 10.1016/S0140-6736(23)00050-8 36641201

[23] Park, T. et al. Vaccines against SARS-CoV-2 variants and future pandemics. *Expert Rev. Vaccines* **21**, 1363–1376 (2022). 10.1080/14760584.2022.2110075 PMC9979704 35924678

[24] Nelde, A. et al. SARS-CoV-2-derived peptides define heterologous and COVID-19-induced T cell recognition. *Nat. Immunol.* **22**, 74–85 (2021). 10.1038/s41590-020-00808-x 32999467

[25] Peng, Y. et al. Broad and strong memory CD4(+) and CD8(+) T cells induced by SARS-CoV-2 in UK convalescent individuals following COVID-19. *Nat. Immunol.* **21**, 1336–1345 (2020). 10.1038/s41590-020-0782-6 PMC7611020 32887977

[26] Schub, D. et al. High levels of SARS-CoV-2-specific T cells with restricted functionality in severe courses of COVID-19. *JCI insight* **5**, e142167 (2020). 10.1172/jci.insight.142167 PMC7605520 32937615

[27] Sette, A. & Crotty, S. Adaptive immunity to SARS-CoV-2 and COVID-19. *Cell* **184**, 861–880 (2021). 10.1016/j.cell.2021.01.007 PMC7803150 33497610

[28] Rydyznski Moderbacher, C. et al. Antigen-specific adaptive immunity to SARS-CoV-2 in acute COVID-19 and associations with age and disease severity. *Cell* **183**, 996–1012 e1019 (2020). 10.1016/j.cell.2020.09.038 PMC7494270 33010815

[29] Brand, I. et al. Broad T cell targeting of structural proteins after SARS-CoV-2 infection: high throughput assessment of T cell reactivity using an automated interferon gamma release assay. *Front. Immunol.* **12**, 688436 (2021). 10.3389/fimmu.2021.688436 PMC8173205 34093595

[30] Bange, E. M. et al. CD8(+) T cells contribute to survival in patients with COVID-19 and hematologic cancer. *Nat. Med.* **27**, 1280–1289 (2021). 10.1038/s41591-021-01386-7 PMC8291091 34017137

[31] Shi, J. et al. A T cell-based SARS-CoV-2 spike protein vaccine provides protection without antibodies. *JCI Insight* **9**, e155789 (2024). 10.1172/jci.insight.155789 PMC10972590 38456504

[32] Zhao, J. et al. Airway memory CD4(+) T cells mediate protective immunity against emerging respiratory coronaviruses. *Immunity* **44**, 1379–1391 (2016). 10.1016/j.immuni.2016.05.006 PMC4917442 27287409

[33] Swadling, L. et al. Pre-existing polymerase-specific T cells expand in abortive seronegative SARS-CoV-2. *Nature* **601**, 110–117 (2022). 10.1038/s41586-021-04186-8 PMC8732273 34758478

[34] Diniz, M. O. et al. Airway-resident T cells from unexposed individuals cross-recognize SARS-CoV-2. *Nat. Immunol.* **23**, 1324–1329 (2022). 10.1038/s41590-022-01292-1 PMC9477726 36038709

[35] Kundu, R. et al. Cross-reactive memory T cells associate with protection against SARS-CoV-2 infection in COVID-19 contacts. *Nat. Commun.* **13**, 80 (2022). 10.1038/s41467-021-27674-x PMC8748880 35013199

[36] Mitsi, E. et al. Respiratory mucosal immune memory to SARS-CoV-2 after infection and vaccination. *Nat. Commun.* **14**, 6815 (2023). 10.1038/s41467-023-42433-w PMC10603102 37884506

[37] Yan, L. et al. Architecture of a SARS-CoV-2 mini replication and transcription complex. *Nat. Commun.* **11**, 5874 (2020). 10.1038/s41467-020-19770-1 PMC7675986 33208736

[38] Finkel, Y. et al. The coding capacity of SARS-CoV-2. *Nature* **589**, 125–130 (2021). 10.1038/s41586-020-2739-1 32906143

[39] Mateus, J. et al. Selective and cross-reactive SARS-CoV-2 T cell epitopes in unexposed humans. *Science* **370**, 89–94 (2020). 10.1126/science.abd3871 PMC7574914 32753554

[40] Primard, C. et al. OVX033, a nucleocapsid-based vaccine candidate, provides broad-spectrum protection against SARS-CoV-2 variants in a hamster challenge model. *Front. Immunol.* **14**, 1188605 (2023). 10.3389/fimmu.2023.1188605 PMC10319154 37409116

[41] Coulon, P. G. et al. High frequencies of alpha common cold coronavirus/SARS-CoV-2 cross-reactive functional CD4(+) and CD8(+) memory T cells are associated with protection from symptomatic and fatal SARS-CoV-2 infections in unvaccinated COVID-19 patients. *Front. Immunol.* **15**, 1343716 (2024). 10.3389/fimmu.2024.1343716 PMC11007208 38605956

[42] Pardi, N., Muramatsu, H., Weissman, D. & Kariko, K. In vitro transcription of long RNA containing modified nucleosides. *Methods Mol. Biol.* **969**, 29–42 (2013). 10.1007/978-1-62703-260-5_2 23296925

[43] Zhang, Y. et al. A highly efficacious live attenuated mumps virus-based SARS-CoV-2 vaccine candidate expressing a six-proline stabilized prefusion spike. *Proc. Natl. Acad. Sci. USA* **119**, e2201616119 (2022). 10.1073/pnas.2201616119 PMC9388148 35895717

[44] Lu, M. et al. SARS-CoV-2 prefusion spike protein stabilized by six rather than two prolines is more potent for inducing antibodies that neutralize viral variants of concern. *Proc. Natl. Acad. Sci. USA* **119**, e2110105119 (2022). 10.1073/pnas.2110105119 PMC9436349 35994646

[45] Maier, M. A. et al. Biodegradable lipids enabling rapidly eliminated lipid nanoparticles for systemic delivery of RNAi therapeutics. *Mol. Ther.* **21**, 1570–1578 (2013). 10.1038/mt.2013.124 PMC3734658 23799535

[46] Sia, S. F. et al. Pathogenesis and transmission of SARS-CoV-2 in golden hamsters. *Nature* **583**, 834–838 (2020). 10.1038/s41586-020-2342-5 PMC7394720 32408338

[47] Imai, M. et al. Syrian hamsters as a small animal model for SARS-CoV-2 infection and countermeasure development. *Proc. Natl. Acad. Sci. USA* **117**, 16587–16595 (2020). 10.1073/pnas.2009799117 PMC7368255 32571934

[48] Dowall, S. et al. Development of a hamster natural transmission model of SARS-CoV-2 infection. *Viruses* **13**, 2251 (2021). 10.3390/v13112251 PMC8625437 34835057

[49] Monchatre-Leroy, E. et al. Hamster and ferret experimental infection with intranasal low dose of a single strain of SARS-CoV-2. *J. Gen. Virol.* **102**, 001567 (2021). 10.1099/jgv.0.001567 PMC8515860 33612147

[50] Chu, H., Chan, J. F. & Yuen, K. Y. Animal models in SARS-CoV-2 research. *Nat. Methods* **19**, 392–394 (2022). 10.1038/s41592-022-01447-w 35396468

[51] Hajnik, R. L. et al. Dual spike and nucleocapsid mRNA vaccination confer protection against SARS-CoV-2 Omicron and Delta variants in preclinical models. *Sci. Transl. Med.* **14**, eabq1945 (2022). 10.1126/scitranslmed.abq1945 PMC9926941 36103514

[52] Desmecht, S. et al. Kinetics and persistence of the cellular and humoral immune responses to BNT162b2 mRNA vaccine in SARS-CoV-2-naive and -experienced subjects: impact of booster dose and breakthrough infections. *Front. Immunol.* **13**, 863554 (2022). 10.3389/fimmu.2022.863554 PMC9195074 35711445

[53] Liu, H. et al. Development of optimized drug-like small molecule inhibitors of the SARS-CoV-2 3CL protease for treatment of COVID-19. *Nat. Commun.* **13**, 1891 (2022). 10.1038/s41467-022-29413-2 PMC8989888 35393402

[54] Kumar, S., Thambiraja, T. S., Karuppanan, K. & Subramaniam, G. Omicron and Delta variant of SARS-CoV-2: A comparative computational study of spike protein. *J. Med. Virol.* **94**, 1641–1649 (2022). 10.1002/jmv.27526 34914115

[55] Chakraborty, D. et al. Stabilizing prefusion SARS-CoV-2 spike by destabilizing the postfusion conformation. *Vaccines* **13**, 315 (2025). 10.3390/vaccines13030315 PMC11946859 40266205

[56] Rutten, L. et al. Impact of SARS-CoV-2 spike stability and RBD exposure on antigenicity and immunogenicity. *Sci. Rep.* **14**, 5735 (2024). 10.1038/s41598-024-56293-x PMC10923862 38459086

[57] Vabret, N. et al. Immunology of COVID-19: current state of the science. *Immunity* **52**, 910–941 (2020). 10.1016/j.immuni.2020.05.002 PMC7200337 32505227

[58] Weisblum, Y. et al. Escape from neutralizing antibodies by SARS-CoV-2 spike protein variants. *eLife* **9**, e61312 (2020). 10.7554/eLife.61312 PMC7723407 33112236

[59] Xie, X. et al. Neutralization of SARS-CoV-2 spike 69/70 deletion, E484K and N501Y variants by BNT162b2 vaccine-elicited sera. *Nat. Med.* **27**, 620–621 (2021). 10.1038/s41591-021-01270-4 33558724

[60] Boppana, S. et al. SARS-CoV-2-specific circulating T follicular helper cells correlate with neutralizing antibodies and increase during early convalescence. *PLoS Pathog.* **17**, e1009761 (2021). 10.1371/journal.ppat.1009761 PMC8318272 34270631

[61] Yuzefpolskiy, Y. et al. Cutting edge: effect of disease-modifying therapies on SARS-CoV-2 vaccine-induced immune responses in multiple sclerosis patients. *J. Immunol.* **208**, 1519–1524 (2022). 10.4049/jimmunol.2101142 35288472

[62] Eser, T. M. et al. Nucleocapsid-specific T cell responses associate with control of SARS-CoV-2 in the upper airways before seroconversion. *Nat. Commun.* **14**, 2952 (2023). 10.1038/s41467-023-38020-8 PMC10209201 37225706

[63] Xu, Z. et al. Pathological findings of COVID-19 associated with acute respiratory distress syndrome. *Lancet Respir. Med.* **8**, 420–422 (2020). 10.1016/S2213-2600(20)30076-X PMC7164771 32085846

[64] Ackermann, M. et al. Pulmonary vascular endothelialitis, thrombosis, and angiogenesis in Covid-19. *N. Engl. J. Med.* **383**, 120–128 (2020). 10.1056/NEJMoa2015432 PMC7412750 32437596

[65] Tian, S. et al. Pulmonary pathology of early-phase 2019 novel coronavirus (COVID-19) pneumonia in two patients with lung cancer. *J. Thorac. Oncol.* **15**, 700–704 (2020). 10.1016/j.jtho.2020.02.010 PMC7128866 32114094

[66] Menter, T. et al. Postmortem examination of COVID-19 patients reveals diffuse alveolar damage with severe capillary congestion and variegated findings in lungs and other organs suggesting vascular dysfunction. *Histopathology* **77**, 198–209 (2020). 10.1111/his.14134 PMC7496150 32364264

[67] Hong, W. et al. A mouse model for SARS-CoV-2-induced acute respiratory distress syndrome. *Signal Transduct. Target. Ther.* **6**, 1 (2021). 10.1038/s41392-020-00451-w PMC7775436 33384407

[68] Winkler, E. S. et al. SARS-CoV-2 infection of human ACE2-transgenic mice causes severe lung inflammation and impaired function. *Nat. Immunol.* **21**, 1327–1335 (2020). 10.1038/s41590-020-0778-2 PMC7578095 32839612

[69] Leist, S. R. et al. A mouse-adapted SARS-CoV-2 induces acute lung injury and mortality in standard laboratory mice. *Cell* **183**, 1070–1085.e12 (2020). 10.1016/j.cell.2020.09.050 PMC7510428 33031744

[70] Amruta, N. et al. Mouse adapted SARS-CoV-2 (MA10) viral infection induces neuroinflammation in standard laboratory mice. *Viruses* **15**, 114 (2022). 10.3390/v15010114 PMC9863644 36680154

[71] Allnoch, L. et al. Vascular inflammation is associated with loss of aquaporin 1 expression on endothelial cells and increased fluid leakage in SARS-CoV-2 infected golden Syrian hamsters. *Viruses* **13**, 639 (2021). 10.3390/v13040639 PMC8069375 33918079

[72] Chan, J. F. et al. Simulation of the clinical and pathological manifestations of coronavirus disease 2019 (COVID-19) in a golden Syrian hamster model: implications for disease pathogenesis and transmissibility. *Clin. Infect. Dis.* **71**, 2428–2446 (2020). 10.1093/cid/ciaa325 PMC7184405 32215622

[73] Boudewijns, R. et al. STAT2 signaling restricts viral dissemination but drives severe pneumonia in SARS-CoV-2 infected hamsters. *Nat. Commun.* **11**, 5838 (2020). 10.1038/s41467-020-19684-y PMC7672082 33203860

[74] Batah, S. S. & Fabro, A. T. Pulmonary pathology of ARDS in COVID-19: a pathological review for clinicians. *Respir. Med.* **176**, 106239 (2021). 10.1016/j.rmed.2020.106239 PMC7674971 33246294

[75] Schaefer, I. M. et al. In situ detection of SARS-CoV-2 in lungs and airways of patients with COVID-19. *Mod. Pathol.* **33**, 2104–2114 (2020). 10.1038/s41379-020-0595-z PMC7304376 32561849

[76] Borczuk, A. C. et al. COVID-19 pulmonary pathology: a multi-institutional autopsy cohort from Italy and New York City. *Mod. Pathol.* **33**, 2156–2168 (2020). 10.1038/s41379-020-00661-1 PMC7463226 32879413

[77] Cummings, M. J. et al. Epidemiology, clinical course, and outcomes of critically ill adults with COVID-19 in New York City: a prospective cohort study. *Lancet* **395**, 1763–1770 (2020). 10.1016/S0140-6736(20)31189-2 PMC7237188 32442528

[78] Liu, Y. et al. Delta spike P681R mutation enhances SARS-CoV-2 fitness over Alpha variant. *Cell Rep.* **39**, 110829 (2022). 10.1016/j.celrep.2022.110829 PMC9050581 35550680

[79] Tamura, T. et al. Virological characteristics of the SARS-CoV-2 Omicron XBB.1.5 variant. *Nat. Commun.* **15**, 1176 (2024). 10.1038/s41467-024-45274-3 PMC10853506 38332154

[80] Shuai, H. et al. Attenuated replication and pathogenicity of SARS-CoV-2 B.1.1.529 Omicron. *Nature* **603**, 693–699 (2022). 10.1038/s41586-022-04442-5 35062016

[81] Bouvet, M. et al. RNA 3’-end mismatch excision by the severe acute respiratory syndrome coronavirus nonstructural protein nsp10/nsp14 exoribonuclease complex. *Proc. Natl. Acad. Sci. USA* **109**, 9372–9377 (2012). 10.1073/pnas.1201130109 PMC3386072 22635272

[82] Hsu, J. C., Laurent-Rolle, M., Pawlak, J. B., Wilen, C. B. & Cresswell, P. Translational shutdown and evasion of the innate immune response by SARS-CoV-2 NSP14 protein. *Proc. Natl. Acad. Sci. USA* **118**, e2101161118 (2021). 10.1073/pnas.2101161118 PMC8214666 34045361

[83] Ferron, F. et al. Structural and molecular basis of mismatch correction and ribavirin excision from coronavirus RNA. *Proc. Natl. Acad. Sci. USA* **115**, E162–E171 (2018). 10.1073/pnas.1718806115 PMC5777078 29279395

[84] Le Bert, N. et al. Highly functional virus-specific cellular immune response in asymptomatic SARS-CoV-2 infection. *J. Exp. Med.* **218**, e20202617 (2021). 10.1084/jem.20202617 PMC7927662 33646265

[85] Chen, Y. et al. Functional screen reveals SARS coronavirus nonstructural protein nsp14 as a novel cap N7 methyltransferase. *Proc. Natl. Acad. Sci. USA* **106**, 3484–3489 (2009). 10.1073/pnas.0808790106 PMC2651275 19208801

[86] Zhao, Y. et al. Structural basis for replicase polyprotein cleavage and substrate specificity of main protease from SARS-CoV-2. *Proc. Natl. Acad. Sci. USA* **119**, e2117142119 (2022). 10.1073/pnas.2117142119 PMC9172370 35380892

[87] van Hemert, M. J. et al. SARS-coronavirus replication/transcription complexes are membrane-protected and need a host factor for activity in vitro. *PLoS Pathog.* **4**, e1000054 (2008). 10.1371/journal.ppat.1000054 PMC2322833 18451981

[88] Hagemeijer, M. C. & de Haan, C. A. Studying the dynamics of coronavirus replicative structures. *Methods Mol. Biol.* **1282**, 261–269 (2015). 10.1007/978-1-4939-2438-7_22 PMC7122990 25720487

[89] Becerra-Artiles, A. et al. Immunopeptidome profiling of human coronavirus OC43-infected cells identifies CD4 T-cell epitopes specific to seasonal coronaviruses or cross-reactive with SARS-CoV-2. *PLoS Pathog.* **19**, e1011032 (2023). 10.1371/journal.ppat.1011032 PMC10409285 37498934

[90] Snedden, C. E. & Lloyd-Smith, J. O. Predicting the presence of infectious virus from PCR data: A meta-analysis of SARS-CoV-2 in non-human primates. *PLoS Pathog.* **20**, e1012171 (2024). 10.1371/journal.ppat.1012171 PMC11081500 38683864

[91] Brant, A. C., Tian, W., Majerciak, V., Yang, W. & Zheng, Z. M. SARS-CoV-2: from its discovery to genome structure, transcription, and replication. *Cell Biosci.* **11**, 136 (2021). 10.1186/s13578-021-00643-z PMC8287290 34281608

[92] Thakur, N., Chakraborty, P., Tufariello, J. M. & Basler, C. F. SARS-CoV-2 Nsp14 binds Tollip and activates pro-inflammatory pathways while downregulating interferon-alpha and interferon-gamma receptors. *mBio* **16**, e0107125 (2025). 10.1128/mbio.01071-25 PMC12345183 40558091

[93] Ghaemi, A. et al. Recombinant COVID-19 vaccine based on recombinant RBD/Nucleoprotein and saponin adjuvant induces long-lasting neutralizing antibodies and cellular immunity. *Front. Immunol.* **13**, 974364 (2022). 10.3389/fimmu.2022.974364 PMC9494508 36159845

[94] Appelberg, S. et al. A universal SARS-CoV DNA vaccine inducing highly cross-reactive neutralizing antibodies and T cells. *EMBO Mol. Med.* **14**, e15821 (2022). 10.15252/emmm.202215821 PMC9538582 35986481

[95] Rice, A. et al. Heterologous saRNA prime, DNA dual-antigen boost SARS-CoV-2 vaccination elicits robust cellular immunogenicity and cross-variant neutralizing antibodies. *Front. Immunol.* **13**, 910136 (2022). 10.3389/fimmu.2022.910136 PMC9335885 35911728

[96] Khan, M. S. et al. Adenovirus-vectored SARS-CoV-2 vaccine expressing S1-N fusion protein. *Antib. Ther.* **5**, 177–191 (2022). 10.1093/abt/tbac015 PMC9372896 35967905

[97] Hasanpourghadi, M. et al. Heterologous chimpanzee adenovirus vector immunizations for SARS-CoV-2 spike and nucleocapsid protect hamsters against COVID-19. *Microbes Infect.* **25**, 105082 (2023). 10.1016/j.micinf.2022.105082 PMC9758783 36539010

[98] Chiuppesi, F. et al. Safety and immunogenicity of a synthetic multiantigen modified vaccinia virus Ankara-based COVID-19 vaccine (COH04S1): an open-label and randomised, phase 1 trial. *Lancet Microbe* **3**, e252–e264 (2022). 10.1016/S2666-5247(22)00027-1 PMC8906816 35287430

[99] Zayou, L. et al. Dynamics of spike-specific neutralizing antibodies across five-year emerging SARS-CoV-2 variants of concern reveal conserved epitopes that protect against severe COVID-19. *Front. Immunol.* **16**, 1503954 (2025). 10.3389/fimmu.2025.1503954 PMC11876060 40040708

[100] Prakash, S. et al. A broad-spectrum multi-antigen mRNA/LNP-based pan-coronavirus vaccine induced potent cross-protective immunity against infection and disease caused by highly pathogenic and heavily spike-mutated SARS-CoV-2 variants of concern in the Syrian hamster model. Preprint at *bioRxiv* 10.1101/2024.02.14.580225 (2024).

[101] Rappaport, A. R. et al. Low-dose self-amplifying mRNA COVID-19 vaccine drives strong protective immunity in non-human primates against SARS-CoV-2 infection. *Nat. Commun.* **13**, 3289 (2022). 10.1038/s41467-022-31005-z PMC9173840 35672369

[102] Arieta, C. M. et al. The T-cell-directed vaccine BNT162b4 encoding conserved non-spike antigens protects animals from severe SARS-CoV-2 infection. *Cell* **186**, 2392–2409.e21 (2023). 10.1016/j.cell.2023.04.007 PMC10099181 37164012

[103] Dervillez, X. et al. “Asymptomatic” HLA-A*02:01-restricted epitopes from herpes simplex virus glycoprotein B preferentially recall polyfunctional CD8+ T cells from seropositive asymptomatic individuals and protect HLA transgenic mice against ocular herpes. *J. Immunol.* **191**, 5124–5138 (2013). 10.4049/jimmunol.1301415 PMC4057607 24101547

[104] Boucherma, R. et al. HLA-A*01:03, HLA-A*24:02, HLA-B*08:01, HLA-B*27:05, HLA-B*35:01, HLA-B*44:02, and HLA-C*07:01 monochain transgenic/H-2 class I null mice: novel versatile preclinical models of human T cell responses. *J. Immunol.* **191**, 583–593 (2013). 10.4049/jimmunol.1300483 PMC4057605 23776170

[105] Sette, A. & Sidney, J. Nine major HLA class I supertypes account for the vast preponderance of HLA-A and -B polymorphism. *Immunogenetics* **50**, 201–212 (1999). 10.1007/s002510050594 10602880

[106] Sette, A. & Sidney, J. HLA supertypes and supermotifs: a functional perspective on HLA polymorphism. *Curr. Opin. Immunol.* **10**, 478–482 (1998). 10.1016/s0952-7915(98)80124-6 9722926

[107] Hertz, T. & Yanover, C. Identifying HLA supertypes by learning distance functions. *Bioinformatics* **23**, e148–e155 (2007). 10.1093/Bioinformatics/btl324 17237084

[108] Bao, L. et al. The pathogenicity of SARS-CoV-2 in hACE2 transgenic mice. *Nature* **583**, 830–833 (2020). 10.1038/s41586-020-2312-y 32380511

[109] Zhang, C., Wu, Z., Li, J. W., Zhao, H. & Wang, G. Q. The cytokine release syndrome (CRS) of severe COVID-19 and Interleukin-6 receptor (IL-6R) antagonist Tocilizumab may be the key to reduce the mortality. *Int. J. Antimicrob. Agents* **55**, 105954 (2020). 10.1016/j.ijantimicag.2020.105954 PMC7118634 32234467

[110] Vaninov, N. In the eye of the COVID-19 cytokine storm. *Nat. Rev. Immunol.* **20**, 277 (2020). 10.1038/s41577-020-0305-6 PMC7132547 32249847

[111] Ma, J. et al. Potential effect of blood purification therapy in reducing cytokine storm as a late complication of critically ill COVID-19. *Clin. Immunol.* **214**, 108408 (2020). 10.1016/j.clim.2020.108408 PMC7118642 32247038

[112] Henderson, L. A. et al. On the alert for cytokine storm: Immunopathology in COVID-19. *Arthritis Rheumatol.* **72**, 1059–1063 (2020). 10.1002/art.41285 PMC7262347 32293098

[113] Sheahan, T. P. et al. An orally bioavailable broad-spectrum antiviral inhibits SARS-CoV-2 in human airway epithelial cell cultures and multiple coronaviruses in mice. *Sci. Transl. Med.* **12**, eabb5883 (2020). 10.1126/scitranslmed.abb5883 PMC7164393 32253226

[114] Li, K. et al. Single-dose, intranasal immunization with recombinant parainfluenza virus 5 expressing Middle East Respiratory Syndrome Coronavirus (MERS-CoV) spike protein protects mice from fatal MERS-CoV infection. *mBio* **11**, e00554-20 (2020). 10.1128/mBio.00554-20 PMC7157776 32265331

[115] Kim, E. et al. Microneedle array delivered recombinant coronavirus vaccines: immunogenicity and rapid translational development. *EBioMedicine* **55**, 102743 (2020). 10.1016/j.ebiom.2020.102743 PMC7128973 32249203

[116] McCray, P. B. Jr. et al. Lethal infection of K18-hACE2 mice infected with severe acute respiratory syndrome coronavirus. *J. Virol.* **81**, 813–821 (2007). 10.1128/JVI.02012-06 PMC1797474 17079315

[117] Murray, S. M. et al. The impact of pre-existing cross-reactive immunity on SARS-CoV-2 infection and vaccine responses. *Nat. Rev. Immunol.* **23**, 304–316 (2023). 10.1038/s41577-022-00809-x PMC9765363 36539527

[118] Morita, R. et al. COVID-19 relapse associated with SARS-CoV-2 evasion from CD4(+) T-cell recognition in an agammaglobulinemia patient. *iScience* **26**, 106685 (2023). 10.1016/j.isci.2023.106685 PMC10116114 37124420

[119] Sabatino, J. J., Jr et al. Multiple sclerosis therapies differentially affect SARS-CoV-2 vaccine-induced antibody and T cell immunity and function. *JCI Insight* **7**, e156978 (2022). 10.1172/jci.insight.156978 PMC8876469 35030101

[120] Park, H. et al. Longitudinal antibody titers measured after COVID-19 mRNA vaccination can identify individuals at risk for subsequent infection. *Sci. Transl. Med.* **17**, eadv4214 (2025). 10.1126/scitranslmed.adv4214 40961221

[121] Munoz-Fontela, C. et al. Animal models for COVID-19. *Nature* **586**, 509–515 (2020). 10.1038/s41586-020-2787-6 PMC8136862 32967005

[122] Prakash, S. et al. Cross-protection induced by highly conserved human B, CD4(+), and CD8(+) T-cell epitopes-based vaccine against severe infection, disease, and death caused by multiple SARS-CoV-2 variants of concern. *Front. Immunol.* **15**, 1328905 (2024). 10.3389/fimmu.2024.1328905 PMC10839970 38318166

[123] Ma, Y., VanKeulen-Miller, R. & Fenton, O. S. mRNA lipid nanoparticle formulation, characterization and evaluation. *Nat. Protoc.* **20**, 2618–2651 (2025). 10.1038/s41596-024-01134-4 PMC12312782 40069324

[124] Quadiri, A. et al. A spike-based mRNA vaccine encapsulated in phospholipid 1,2-dioleoyl-sn-glycero-3-phosphoethanolamine containing lipid nanoparticles induced potent B- and T-cell responses associated with protection against SARS-CoV-2 infection and COVID-19-like symptoms in hamsters. *Vaccines* **13**, 47 (2025). 10.3390/vaccines13010047 PMC11769137 39852826

[125] Muruato, A. E. et al. A high-throughput neutralizing antibody assay for COVID-19 diagnosis and vaccine evaluation. *Nat. Commun.* **11**, 4059 (2020). 10.1038/s41467-020-17892-0 PMC7426916 32792628

[126] Matute-Bello, G. et al. An official American Thoracic Society workshop report: features and measurements of experimental acute lung injury in animals. *Am. J. Respir. Cell Mol. Biol.* **44**, 725–738 (2011). 10.1165/rcmb.2009-0210ST PMC7328339 21531958

[127] Bi, Z., Hong, W., Yang, J., Lu, S. & Peng, X. Animal models for SARS-CoV-2 infection and pathology. *MedComm* **2**, 548–568 (2021). 10.1002/mco2.98 PMC8662225 34909757

[128] Winkler, E. S. et al. Publisher Correction: SARS-CoV-2 infection of human ACE2-transgenic mice causes severe lung inflammation and impaired function. *Nat. Immunol.* **21**, 1470 (2020). 10.1038/s41590-020-0794-2 PMC7464056 32879513

