# Supplementary material for: Multi-antigen coronavirus vaccine induces potent and durable cross-protection against multiple SARS-CoV-2 variants
Source: NPJ Vaccines. 2026 Aug 18;11:185. doi: 10.1038/s41541-026-01549-y (PMC13638991; doi:10.1038/s41541-026-01549-y)
Supplement: Supplementary file 1 [file 41541_2026_1549_MOESM1_ESM.pdf]

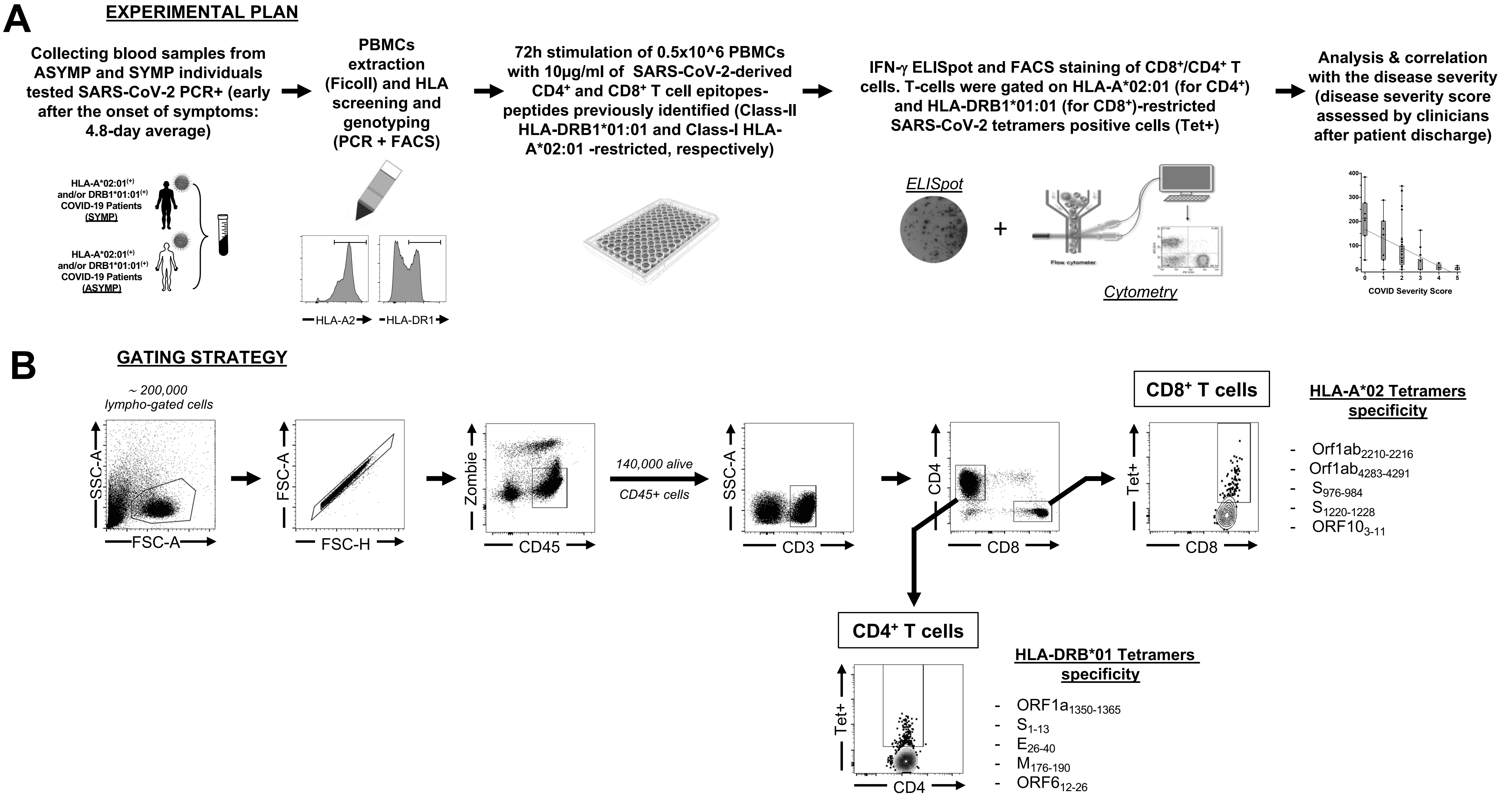
A
EXPERIMENTAL PLAN
Collecting blood samples from ASYMP and SYMP individuals tested SARS-CoV-2 PCR+ (early after the onset of symptoms: 4.8-day average)
PBMCs extraction (Ficoll) and HLA screening and genotyping (PCR + FACS)
72h stimulation of 0.5x10^6 PBMCs with 10µg/ml of SARS-CoV-2-derived CD4+ and CD8+ T cell epitopes-peptides previously identified (Class-II HLA-DRB1*01:01 and Class-I HLA-A*02:01 -restricted, respectively)
IFN-γ ELISpot and FACS staining of CD8+/CD4+ T cells. T-cells were gated on HLA-A*02:01 (for CD4+) and HLA-DRB1*01:01 (for CD8+)-restricted SARS-CoV-2 tetramers positive cells (Tet+)
Analysis & correlation with the disease severity (disease severity score assessed by clinicians after patient discharge)
HLA-A*02:01(+) and/or DRB1*01:01(+) COVID-19 Patients (SYMP)
HLA-A*02:01(+) and/or DRB1*01:01(+) COVID-19 Patients (ASYMP)
HLA-A2
HLA-DR1
ELISpot
+
Flow cytometer
Cytometry
COVID Severity Score
B
GATING STRATEGY
~ 200,000 lympho-gated cells
SSC-A
FSC-A
FSC-A
FSC-H
Zombie
CD45
140,000 alive CD45+ cells
SSC-A
CD3
CD4
CD8
CD8+ T cells
Tet+
CD8
HLA-A*02 Tetramers specificity
- Orf1ab2210-2216
- Orf1ab4283-4291
- S976-984
- S1220-1228
- ORF103-11
CD4+ T cells
Tet+
CD4
HLA-DRB*01 Tetramers specificity
- ORF1a1350-1365
- S1-13
- E26-40
- M176-190
- ORF612-26

**Supplementary Figure 1. Experimental plan and gating strategy:** (A) shows the plan followed for the flow cytometry and ELISpot experiments, including COVID-19 patient genotyping, blood sampling, PBMC extraction, and peptide stimulation. Fresh blood samples were collected from COVID-19 patients during the acute phase of infection (days 3-8) who were admitted to UC Irvine hospitals. For non-admitted patients, fresh blood was collected on day 2 after the onset of COVID-19-like symptoms and when they visited the hospital for a check-up. The average time from the onset of COVID-19 symptoms to PBMC collection was 4.8 days. Fresh PBMCs were used in this study because they generally have higher viability and functionality than frozen PBMCs. (B) shows the gating strategy used to analyze the flow cytometry data presented in this study.

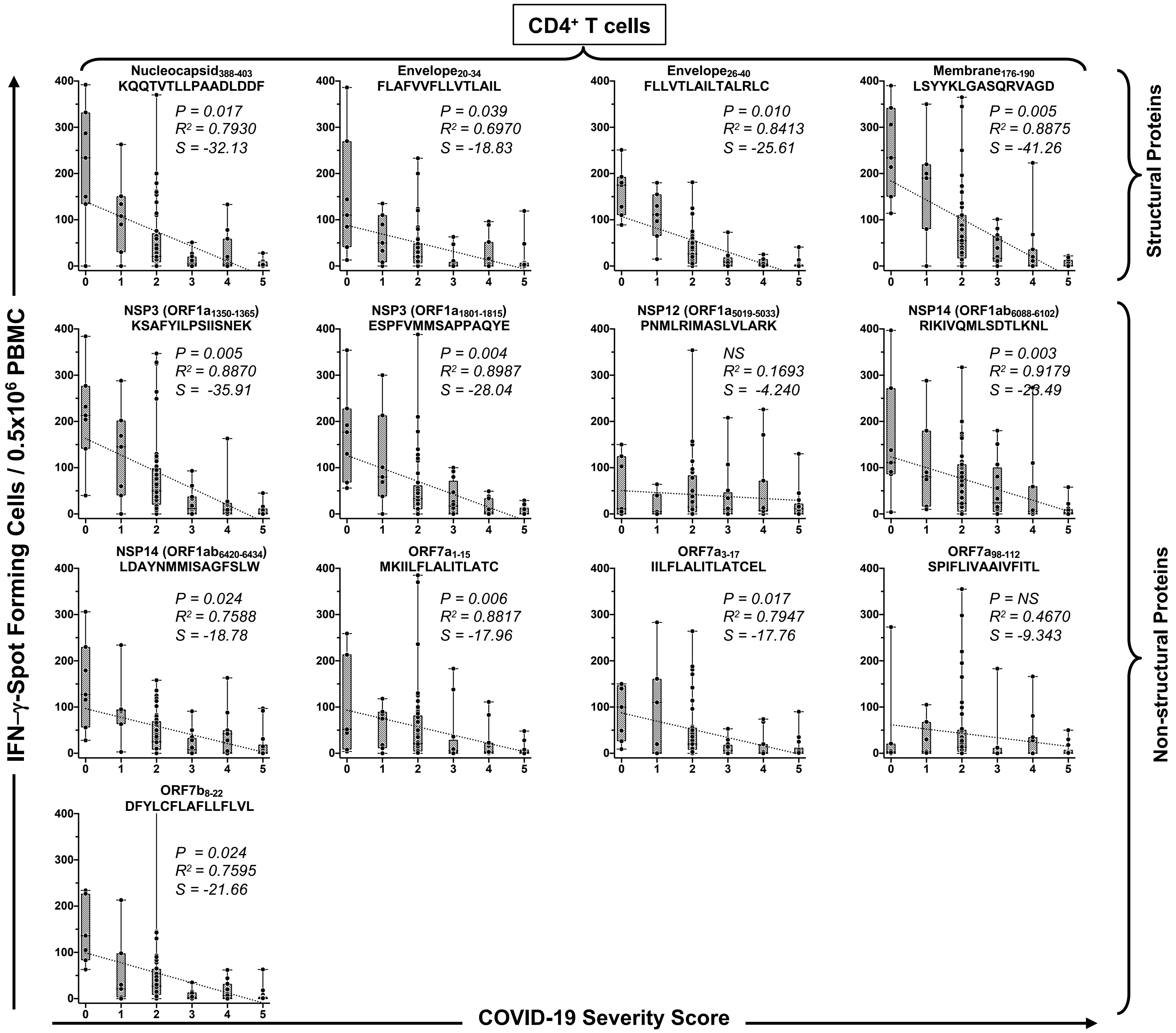
CD4+ T cells
Structural Proteins
Non-structural Proteins
IFN-γ-Spot Forming Cells / 0.5x10^6 PBMC
COVID-19 Severity Score
Nucleocapsid388-403
KQQTVTLLPAADLDDF
P = 0.017
R2 = 0.7930
S = -32.13
Envelope20-34
FLAFVVFLLVTLAIL
P = 0.039
R2 = 0.6970
S = -18.83
Envelope26-40
FLLVTLAILTALRLC
P = 0.010
R2 = 0.8413
S = -25.61
Membrane176-190
LSYYKLGASQRVAGD
P = 0.005
R2 = 0.8875
S = -41.26
NSP3 (ORF1a1350-1365)
KSAFYILPSIISNEK
P = 0.005
R2 = 0.8870
S = -35.91
NSP3 (ORF1a1801-1815)
ESPFVMMSAPPAQYE
P = 0.004
R2 = 0.8987
S = -28.04
NSP12 (ORF1a5019-5033)
PNMLRIMASLVLARK
NS
R2 = 0.1693
S = -4.240
NSP14 (ORF1ab6088-6102)
RIKIVQMLSDTLKNL
P = 0.003
R2 = 0.9179
S = -23.49
NSP14 (ORF1ab6420-6434)
LDAYNMMISAGFSLW
P = 0.024
R2 = 0.7588
S = -18.78
ORF7a1-15
MKIILFLALITLATC
P = 0.006
R2 = 0.8817
S = -17.96
ORF7a3-17
IILFLALITLATCEL
P = 0.017
R2 = 0.7947
S = -17.76
ORF7a98-112
SPIFLIVAAIVFITL
P = NS
R2 = 0.4670
S = -9.343
ORF7b8-22
DFYLCFLAFLLFLVL
P = 0.024
R2 = 0.7595
S = -21.66
0
100
200
300
400
0 1 2 3 4 5

**Supplementary Figure 2. IFN-g-producing CD4$^+$ T cell responses to highly conserved antigens in unvaccinated COVID-19 patients with various degrees of disease severity:** COVID-19 patients (*n* = 71) were divided into six groups based on disease severity, scored 0 to 5, as described in *Materials and Methods.* The magnitude of the 13 individual cross-reactive CD4$^+$ T cell epitope peptides that belong to the selected 10 highly conserved antigens (i.e., NSP-2, NSP-3, NSP-4, NSP-5-10, NSP-12, NSP-14, ORF7a/b, Membrane, Envelope, and Nucleoprotein) are shown as the average/mean numbers (± SD) of IFN-g-spot forming cells (SFCs) after CD4$^+$ T cell peptide stimulation. The coefficient of determination ($R^2$) was calculated from the Pearson correlation coefficients. The associated *P*-value and the slope (S) of the best-fitted line (dotted line) were calculated using linear regression analysis and are indicated. The gray-hatched boxes in the correlation graphs extend from the 25$^{th}$ to the 75th percentiles (hinges of the plots), with the median represented as a horizontal line in each box, and the vertical bars extend to the minimum and maximum values. Results are representative of two independent experiments and were considered statistically significant at $P \leq 0.05$ using either the Mann-Whitney test (two groups) or the Kruskal-Wallis test (more than two groups).

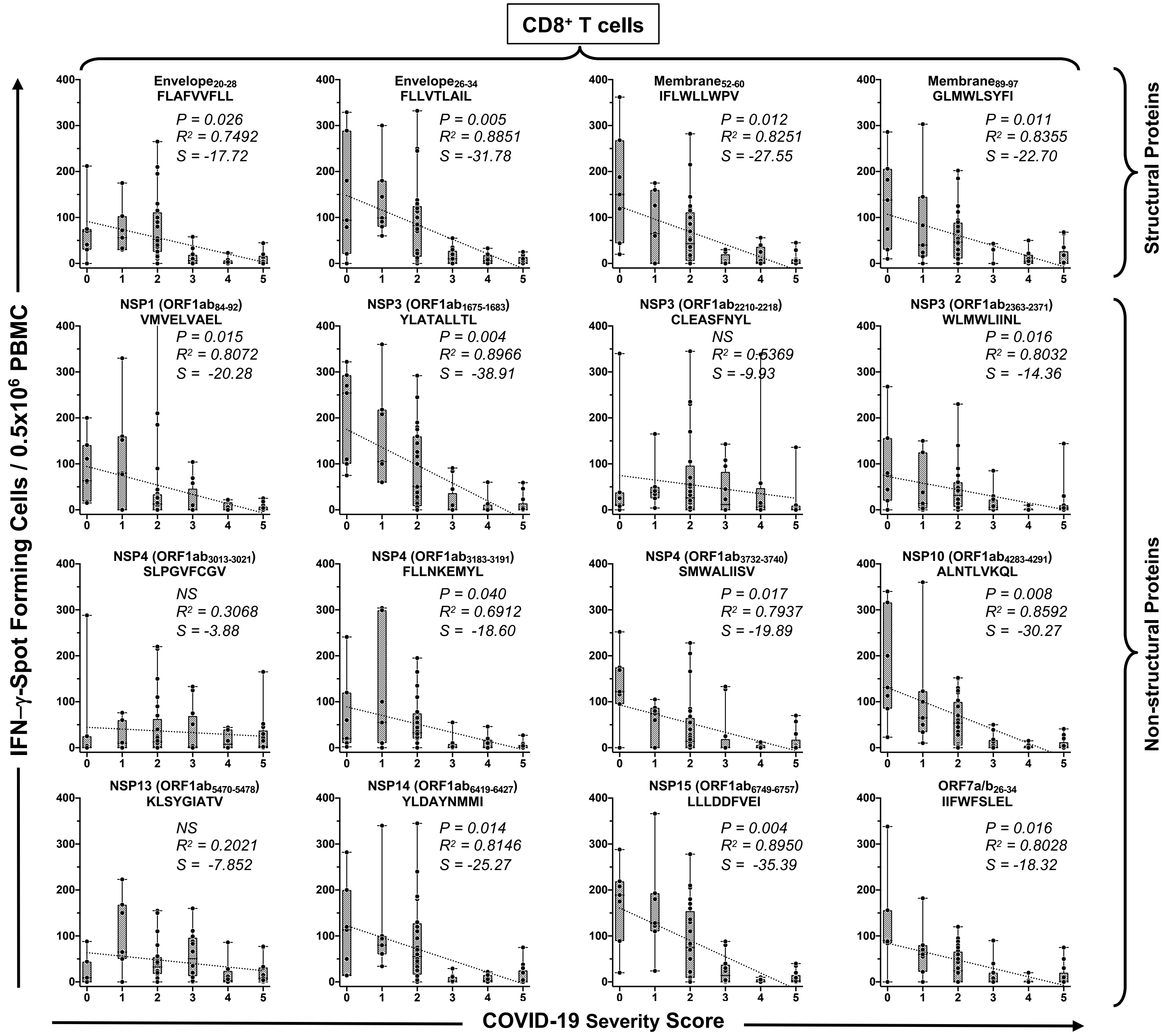
CD8+ T cells
Structural Proteins
Non-structural Proteins
IFN–γ-Spot Forming Cells / 0.5x10^6 PBMC
COVID-19 Severity Score
Envelope20-28 FLAFVVFLL
P = 0.026
R2 = 0.7492
S = -17.72
Envelope26-34 FLLVTLAIL
P = 0.005
R2 = 0.8851
S = -31.78
Membrane52-60 IFLWLLWPV
P = 0.012
R2 = 0.8251
S = -27.55
Membrane89-97 GLMWLSYFI
P = 0.011
R2 = 0.8355
S = -22.70
NSP1 (ORF1ab84-92) VMVELVAEL
P = 0.015
R2 = 0.8072
S = -20.28
NSP3 (ORF1ab1675-1683) YLATALLTL
P = 0.004
R2 = 0.8966
S = -38.91
NSP3 (ORF1ab2210-2218) CLEASFNYL
NS
R2 = 0.5369
S = -9.93
NSP3 (ORF1ab2363-2371) WLMWLIINL
P = 0.016
R2 = 0.8032
S = -14.36
NSP4 (ORF1ab3013-3021) SLPGVFCGV
NS
R2 = 0.3068
S = -3.88
NSP4 (ORF1ab3183-3191) FLLNKEMYL
P = 0.040
R2 = 0.6912
S = -18.60
NSP4 (ORF1ab3732-3740) SMWALIISV
P = 0.017
R2 = 0.7937
S = -19.89
NSP10 (ORF1ab4283-4291) ALNTLVKQL
P = 0.008
R2 = 0.8592
S = -30.27
NSP13 (ORF1ab5470-5478) KLSYGIATV
NS
R2 = 0.2021
S = -7.852
NSP14 (ORF1ab6419-6427) YLDAYNMMI
P = 0.014
R2 = 0.8146
S = -25.27
NSP15 (ORF1ab6749-6757) LLLDDFVEI
P = 0.004
R2 = 0.8950
S = -35.39
ORF7a/b26-34 IIFWFSLEL
P = 0.016
R2 = 0.8028
S = -18.32

**<u>Supplementary Figure 3</u>. IFN-g-producing CD8$^+$ T cell responses to highly conserved antigens in unvaccinated COVID-19 patients with various degrees of disease severity:** COVID-19 patients (*n* = 71) were divided into six groups based on disease severity, scored 0 to 5, as described in *Materials and Methods*. The magnitude of the 16 individual cross-reactive CD8$^+$ T cell epitope peptides that belong to the selected 10 highly conserved antigens (i.e., NSP-2, NSP-3, NSP-4, NSP-5-10, NSP-12, NSP-14, ORF7a/b, Membrane, Envelope, and Nucleoprotein) are shown as the average/mean numbers ($\pm$ SD) of IFN-g-spot forming cells (SFCs) after CD8$^+$ T cell peptide stimulation, The coefficient of determination ($R^2$) was calculated from the Pearson correlation coefficients. The associated *P*-value and the slope (S) of the best-fitted line (dotted line) were calculated using linear regression analysis and are indicated. The gray-hatched boxes in the correlation graphs extend from the 25$^{th}$ to the 75th percentiles (hinges of the plots), with the median represented as a horizontal line in each box, and the vertical bars extend to the minimum and maximum values. Results are representative of two independent experiments and were considered statistically significant at $P \leq 0.05$ using either the Mann-Whitney test (two groups) or the Kruskal-Wallis test (more than two groups).

**Efficient expression of individual Large Sequence Antigen mRNA/LNP vaccines *in vitro* in HEK293 cells**

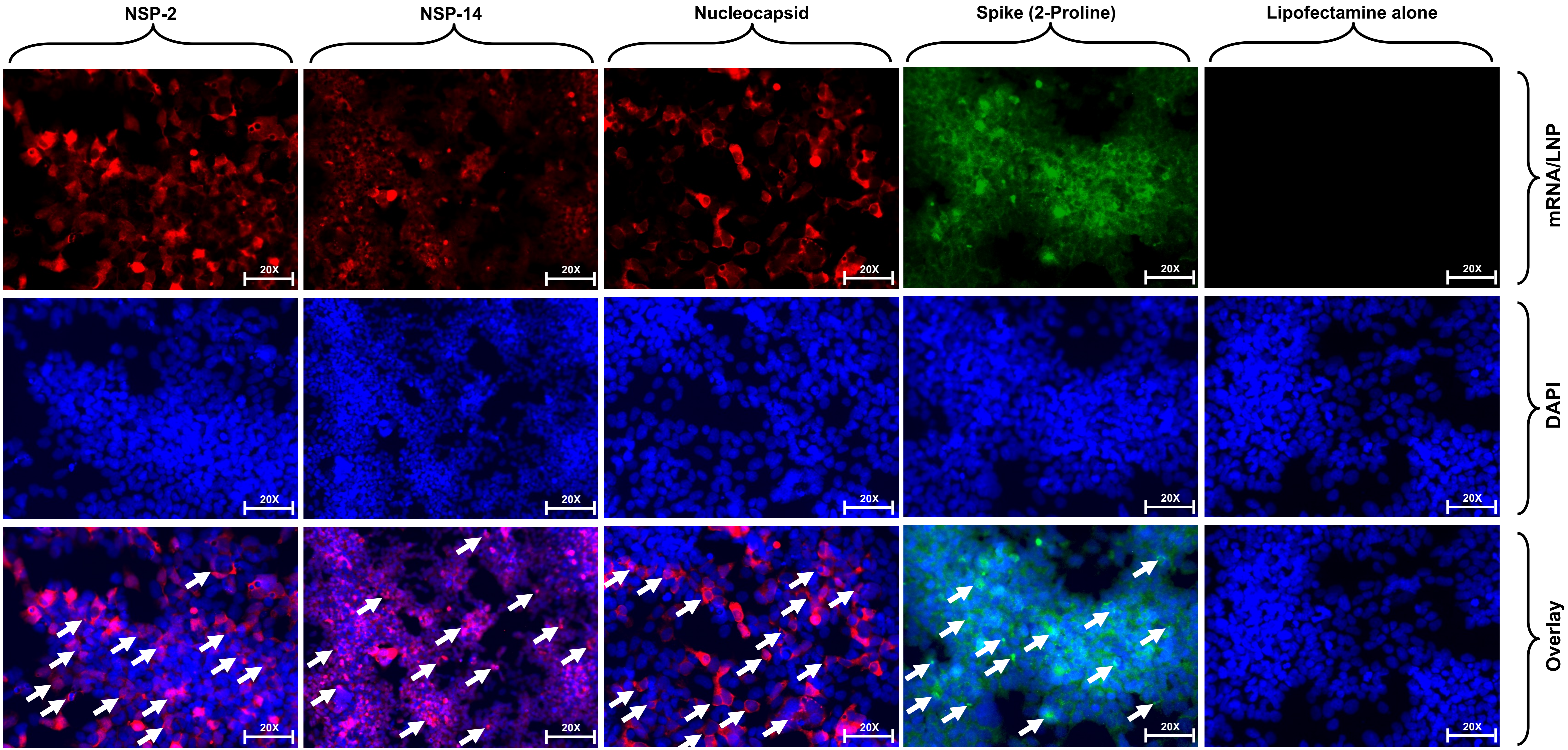

**Supplementary Figure 4. Antigen expression *in vitro* produced by the mRNA-LNP vaccine candidates:** Transfection experiments were performed to ensure that the mRNA-LNP-based T cell antigens are efficiently expressed in the HEK293 cell line. Data show mRNA-LNP vaccine expression alone (NSP-2, NSP-14, and Nucleocapsid in the PE Channel and Spike-2P in the GFP channel). Further, a DAPI-only panel for each of the four antigens is shown, followed by the Overlay data. All images are captured at 20x resolution.

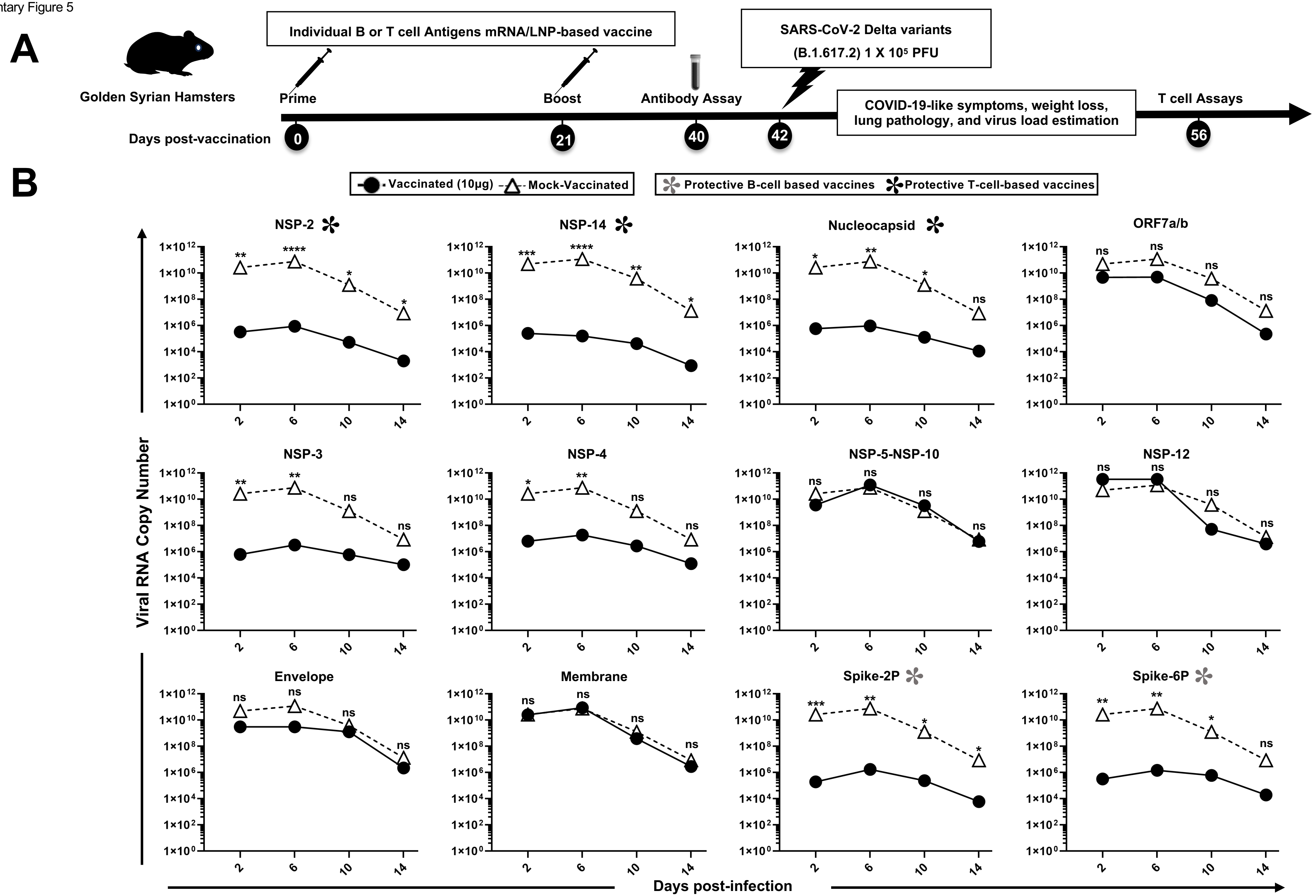
A
Golden Syrian Hamsters
Individual B or T cell Antigens mRNA/LNP-based vaccine
Prime
Boost
Antibody Assay
SARS-CoV-2 Delta variants
(B.1.617.2) 1 X 10⁵ PFU
COVID-19-like symptoms, weight loss, lung pathology, and virus load estimation
T cell Assays
Days post-vaccination
0
21
40
42
56
B
Vaccinated (10µg)
Mock-Vaccinated
Protective B-cell based vaccines
Protective T-cell-based vaccines
NSP-2
NSP-14
Nucleocapsid
ORF7a/b
NSP-3
NSP-4
NSP-5-NSP-10
NSP-12
Envelope
Membrane
Spike-2P
Spike-6P
Viral RNA Copy Number
1×10^12
1×10^10
1×10^8
1×10^6
1×10^4
1×10^2
1×10^0
2
6
10
14
Days post-infection

**<u>Supplementary Figure 5</u>. SARS-CoV-2 viral copy number in golden Syrian hamsters after challenge with the SARS-CoV-2 Delta variant:** (**A**) Experimental plan to screen the vaccine efficacy of the 10 highly conserved T-cell Ags. Male hamsters (*n* = 6 per group) were immunized intramuscularly on day 0 (prime) and day 21 (boost) with 10 mg/dose of the mRNA/LNP-based Coronavirus vaccine expressing each of the 10 highly conserved non-Spike T-cell antigens. Hamsters that received phosphate-buffered saline alone were used as mock-immunized controls (*Saline*, *Mock*, *n* = 6). Three weeks after booster vaccination (day 42), vaccinated and mock-vaccinated hamsters were intranasally challenged (in both nostrils) with 1 × 10^5 pfu of the highly pathogenic SARS-CoV-2 Delta variant (B.1.617.2). (**B**) Shows SARS-CoV-2 viral RNA copy number in hamsters on Days 2, 6, 10, and 14 post-challenge. Hamsters were initially immunized with mRNA/LNP expressing NSP-2, NSP-3, NSP-4, NSP-5-10, NSP-12, NSP-14, ORF7*a*/b, Membrane, Envelope, Nucleoprotein, Spike 2P and Spike 6P at 10 mg/dose. The data represent two independent experiments; the graphed values and bars represent the standard deviation (SD) between the two experiments. Mann-Whitney test (for two groups) or the Kruskal-Wallis test (for more than two groups) was used for statistical analysis. ns $P > 0.05$, * $P < 0.05$, ** $P < 0.01$, *** $P < 0.001$, **** $P < 0.0001$.

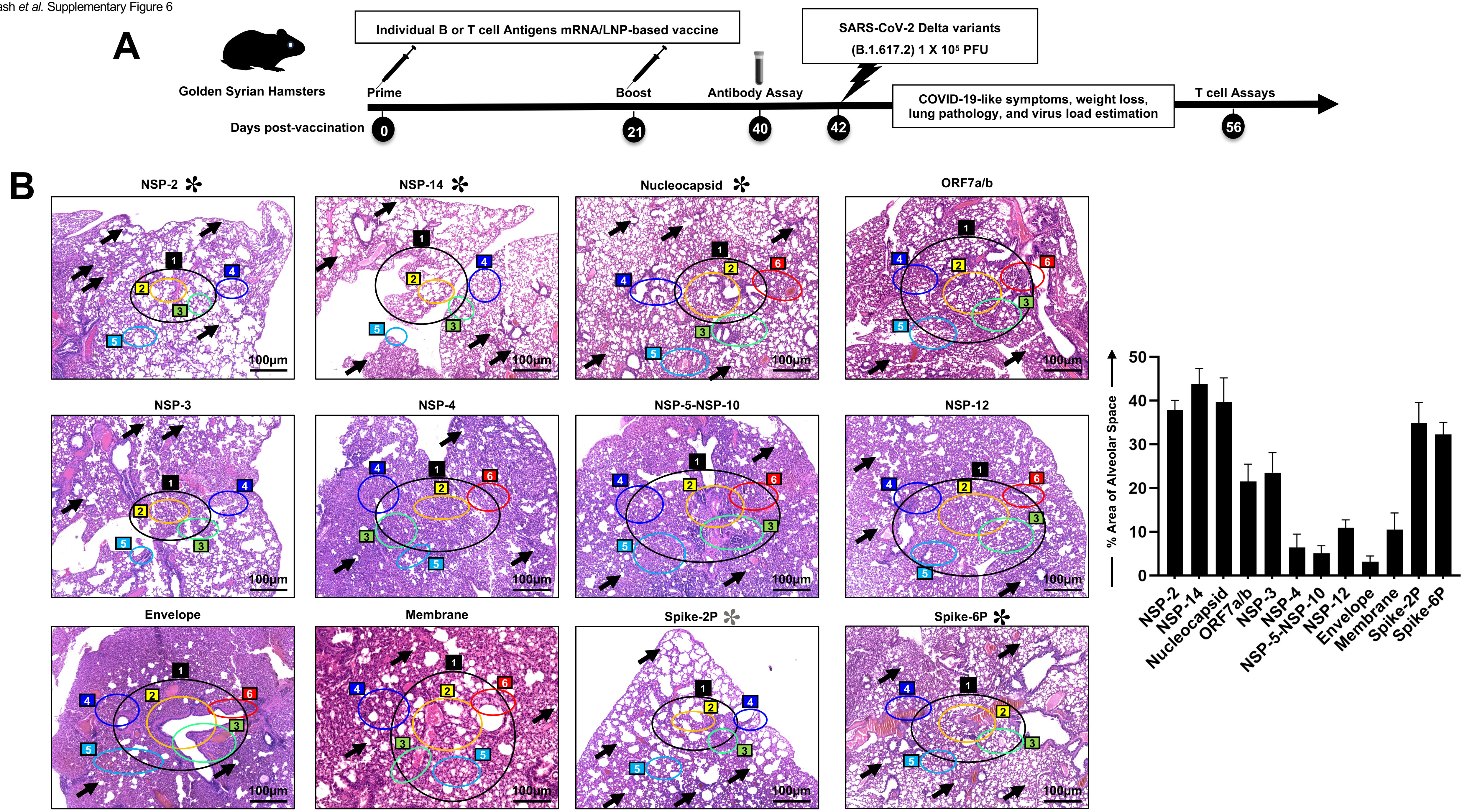
A
Golden Syrian Hamsters
Individual B or T cell Antigens mRNA/LNP-based vaccine
Prime
Boost
Antibody Assay
SARS-CoV-2 Delta variants
(B.1.617.2) 1 X 10⁵ PFU
COVID-19-like symptoms, weight loss, lung pathology, and virus load estimation
T cell Assays
Days post-vaccination
0
21
40
42
56
B
NSP-2
NSP-14
Nucleocapsid
ORF7a/b
NSP-3
NSP-4
NSP-5-NSP-10
NSP-12
Envelope
Membrane
Spike-2P
Spike-6P
100µm
% Area of Alveolar Space
0
10
20
30
40
50
NSP-2
NSP-14
Nucleocapsid
ORF7a/b
NSP-3
NSP-4
NSP-5-NSP-10
NSP-12
Envelope
Membrane
Spike-2P
Spike-6P

**Supplementary Figure 6. Lung pathology in hamsters after challenge with the SARS-CoV-2 Delta variant:** (**A**) Experimental plan to screen the vaccine efficacy of the 10 highly conserved T-cell Ags. Male hamsters (*n* = 6 per group) were immunized intramuscularly on day 0 (prime) and day 21 (boost) with 10 mg/dose of the mRNA/LNP-based Coronavirus vaccine expressing each of the 10 highly conserved non-Spike T-cell antigens. Hamsters that received phosphate-buffered saline alone were used as mock-immunized controls (*Saline*, *Mock*, *n* = 6). Three weeks after booster vaccination (day 42), vaccinated and mock-vaccinated hamsters were intranasally challenged (in both nostrils) with 1 × 10^5 pfu of the highly pathogenic SARS-CoV-2 Delta variant (B.1.617.2). (**B**) Representative images of hematoxylin and eosin (H & E) staining of the lungs harvested on day 14 p.i. are shown. On the H&E-stained lung slides, the following histopathological characteristics are numbered: 1 = diffuse alveolar damage (DAD), assessed as a composite pattern. DAD includes 2 = alveolar septal thickening, 3 = inflammatory cell infiltration, 4 = pneumocyte injury, 5 = intra-alveolar proteinaceous exudate, and 6 = hyaline membrane formation. Reduced COVID-19 lung pathology was observed in hamsters immunized with NSP-2, NSP-14, Nucleocapsid, and the Spike vaccines (Spike-2P, Spike-6P), compared with ORF7a/b, NSP-3, NSP-4, NSP-5-NSP-10, NSP-12, Envelope, and Membrane, as characterized by a higher number of lung vacuoles (marked by black arrows). The image is taken at 4X magnification, and the scale bar is 100 µm.

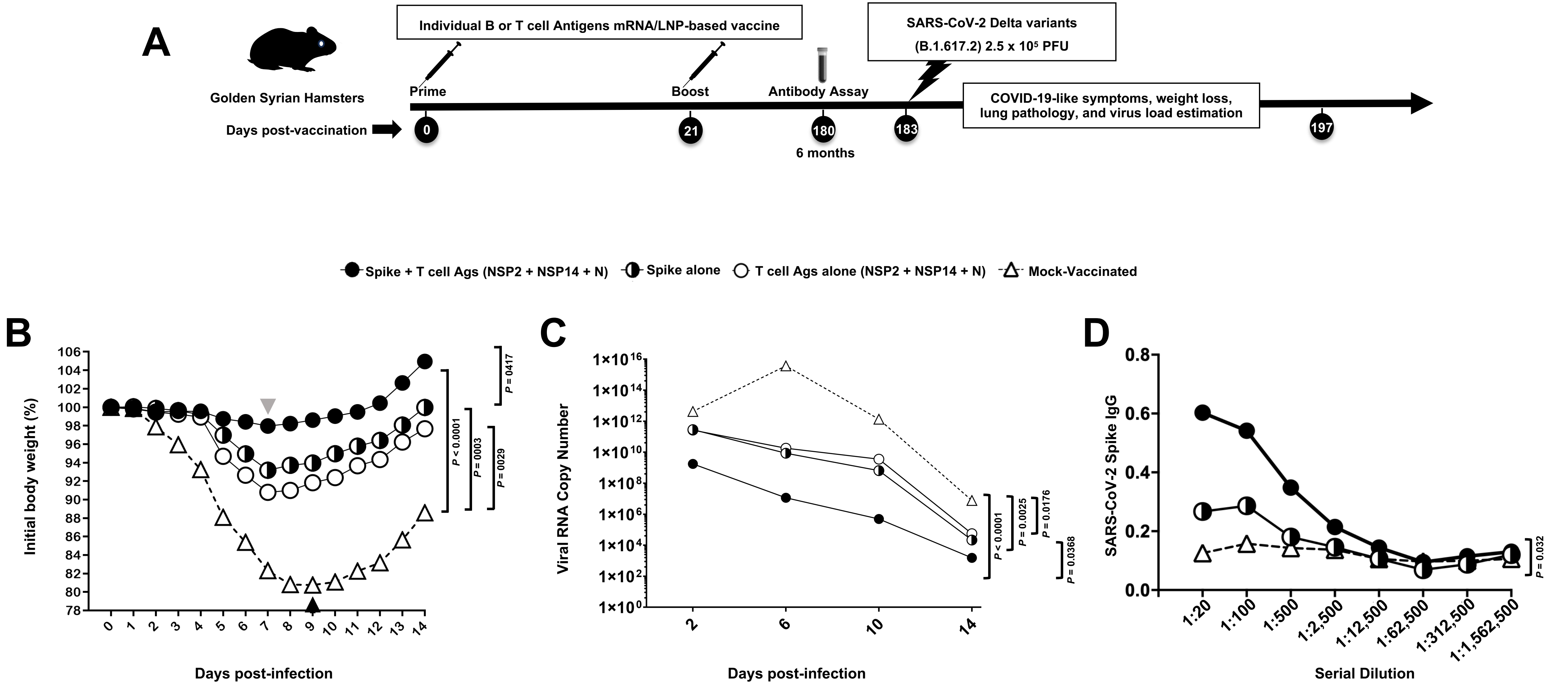
A
Golden Syrian Hamsters
Days post-vaccination
Individual B or T cell Antigens mRNA/LNP-based vaccine
Prime
Boost
Antibody Assay
SARS-CoV-2 Delta variants
(B.1.617.2) 2.5 x 10⁵ PFU
COVID-19-like symptoms, weight loss, lung pathology, and virus load estimation
0
21
180
6 months
183
197
Spike + T cell Ags (NSP2 + NSP14 + N)
Spike alone
T cell Ags alone (NSP2 + NSP14 + N)
Mock-Vaccinated
B
Initial body weight (%)
Days post-infection
P = 0417
P < 0.0001
P = 0003
P = 0029
C
Viral RNA Copy Number
Days post-infection
P < 0.0001
P = 0.0025
P = 0.0176
P = 0.0368
D
SARS-CoV-2 Spike IgG
Serial Dilution
1:20
1:100
1:500
1:2,500
1:12,500
1:62,500
1:312,500
1:1,562,500
P = 0.032

**Supplementary Figure 7. Protection from SARS-CoV-2 challenge at six months following immunization:** (**A**) Male hamsters (*n* = 6 per group) were immunized intramuscularly on day 0 (prime) and day 21 (boost) with 10 mg/dose of the mRNA/LNP-based Coronavirus vaccines. Hamsters that received phosphate-buffered saline alone were used as mock-immunized controls (*Saline*, *Mock*, *n* = 6). Six months after the booster vaccination (day 183), vaccinated and mock-vaccinated hamsters were intranasally challenged (in both nostrils) with $2.5 \times 10^5$ pfu of the highly pathogenic SARS-CoV-2 Delta variant (B.1.617.2). (**B**) Shows percent weight loss following challenge with the Delta variant. (**C**) Shows SARS-CoV-2 viral RNA copy number in hamsters on Days 2, 6, 10, and 14 post-challenge with SARS-CoV-2 Delta (B.1.617.2) variant challenge. The data represent two independent experiments; the graphed values and bars represent the standard deviation (SD) between the two experiments. Mann-Whitney test (for two groups) or the Kruskal-Wallis test (for more than two groups) was used for statistical analysis.

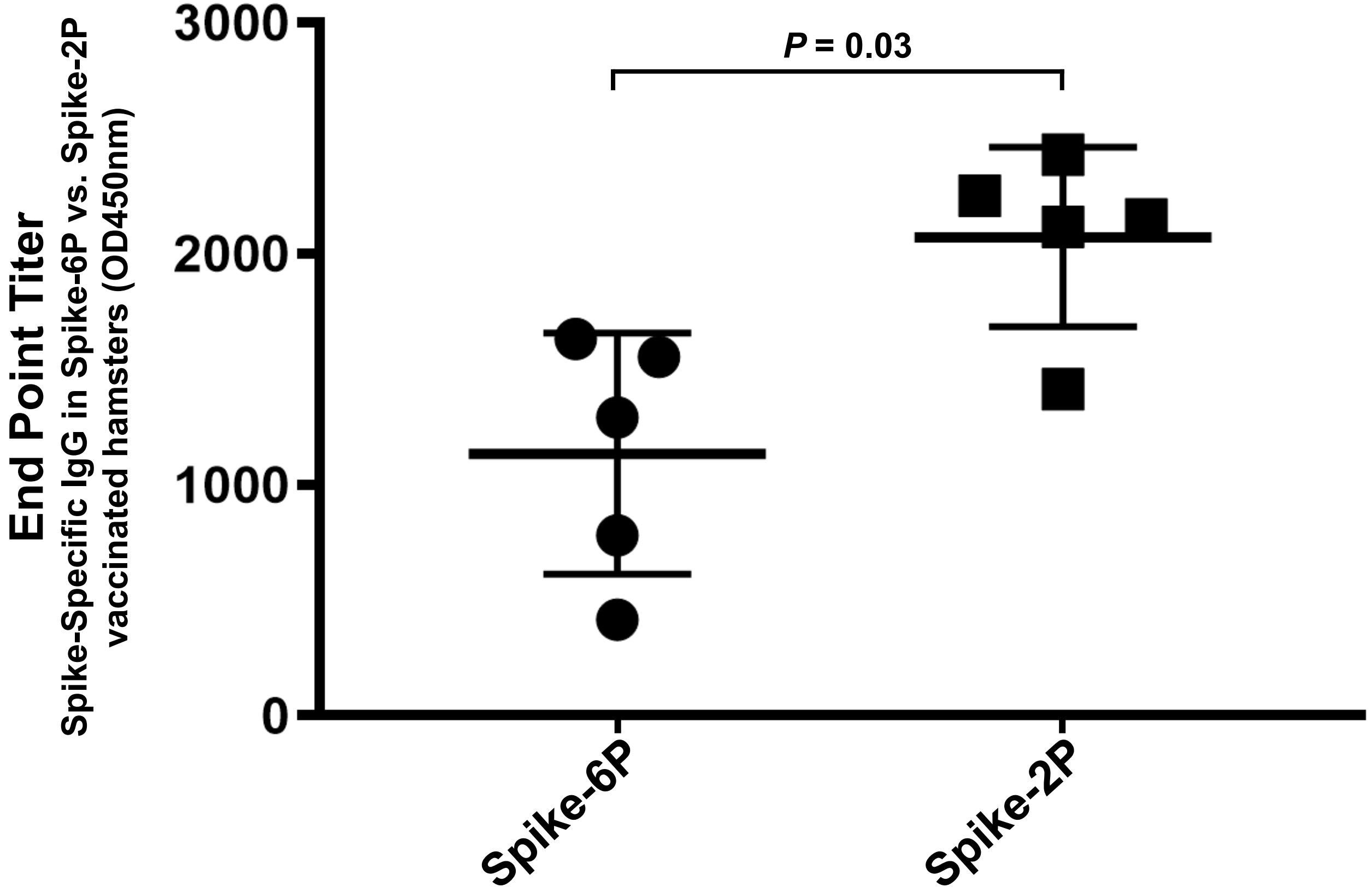
End Point Titer
Spike-Specific IgG in Spike-6P vs. Spike-2P
vaccinated hamsters (OD450nm)
3000
2000
1000
0
P = 0.03
Spike-6P
Spike-2P

**Supplementary Figure 8. Antibody titer in Immunized hamsters:** Endpoint titer showing statistically significant increase in Spike-specific IgG antibody in the serum of hamsters immunized with Spike 2 proline (1µg) compared to Spike 6 proline (1µg). (Blood was collected at day 40 after the first immunization) ELISA plates were coated with 100ng of Spike (S1+S2) from Sino Biological.

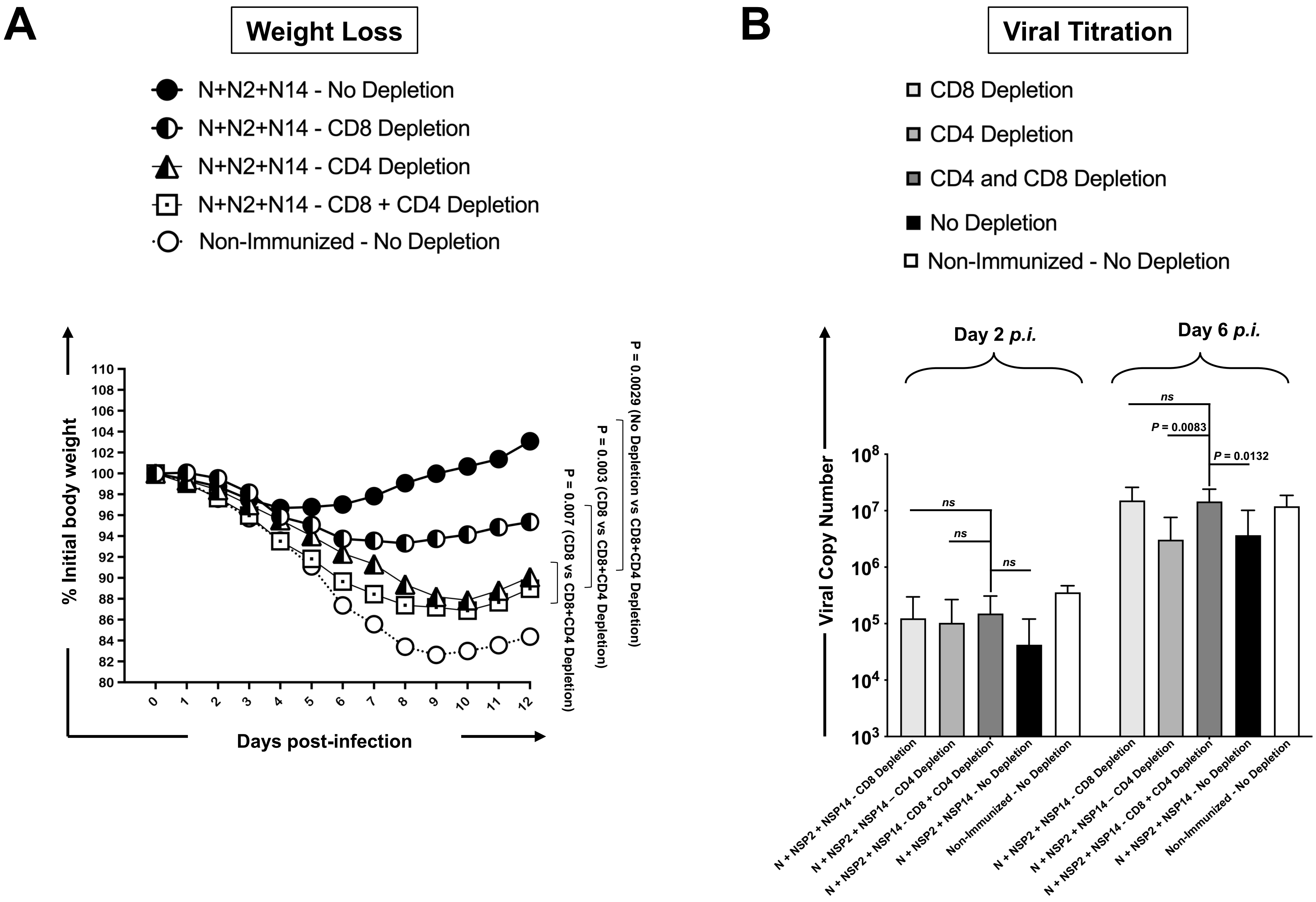
A
Weight Loss
N+N2+N14 - No Depletion
N+N2+N14 - CD8 Depletion
N+N2+N14 - CD4 Depletion
N+N2+N14 - CD8 + CD4 Depletion
Non-Immunized - No Depletion
% Initial body weight
Days post-infection
P = 0.0029 (No Depletion vs CD8+CD4 Depletion)
P = 0.003 (CD8 vs CD8+CD4 Depletion)
P = 0.007 (CD8 vs CD8+CD4 Depletion)
B
Viral Titration
CD8 Depletion
CD4 Depletion
CD4 and CD8 Depletion
No Depletion
Non-Immunized - No Depletion
Day 2 p.i.
Day 6 p.i.
ns
P = 0.0083
P = 0.0132
Viral Copy Number
N + NSP2 + NSP14 - CD8 Depletion
N + NSP2 + NSP14 – CD4 Depletion
N + NSP2 + NSP14 - CD8 + CD4 Depletion
N + NSP2 + NSP14 - No Depletion
Non-Immunized - No Depletion

**<u>Supplementary Figure 9</u>. T cell depletion abrogates protection in SARS-CoV-2 infection:** Rat IgG2b monoclonal antibodies (mAbs) specific for mouse CD4 (Clone Gk1.5) and mouse CD8 (Clone 2.43) were used for in vivo depletion. hACE2 transgenic mice were injected with 300μg of anti-mouse CD4 and/or anti-mouse CD8α mAbs for each dose, given intraperitoneally (i.p.) 4 and 2 days before the challenge with SARS-CoV-2 B.617.2 variant (Delta). Mice were subsequently followed till Day 14 post-infection to monitor Physical estimation (Weight loss, and Viral Titration) comparison of mice immunized with combination of Nucleocapsid + NSP2 + NSP14 (at 1ug each) and subjected to CD8, CD4 and CD4+CD8 T-cell depletion vs immunized mice in which the T cells were not depleted. Immunized mice infected with SARS-CoV-2 Delta variant (at 1 x $10^5$ pfu).
